# NlpI-mediated modulation of outer membrane vesicle production through peptidoglycan dynamics in *Escherichia coli*

**DOI:** 10.1002/mbo3.244

**Published:** 2015-03-08

**Authors:** Carmen Schwechheimer, Daniel L Rodriguez, Meta J Kuehn

**Affiliations:** Department of Biochemistry, Duke University Medical CenterDurham, North Carolina, 27710

**Keywords:** Endopeptidase, Gram-negative bacteria, Lpp, murein, PBP4, secretion, Spr

## Abstract

Outer membrane vesicles (OMVs) are ubiquitously secreted from the outer membrane (OM) of Gram-negative bacteria. These heterogeneous structures are composed of OM filled with periplasmic content from the site of budding. By analyzing mutants that have vesicle production phenotypes, we can gain insight into the mechanism of OMV budding in wild-type cells, which has thus far remained elusive. In this study, we present data demonstrating that the hypervesiculation phenotype of the *nlpI* deletion mutant of *Escherichia coli* correlates with changes in peptidoglycan (PG) dynamics. Our data indicate that in stationary phase cultures the *nlpI* mutant exhibits increased PG synthesis that is dependent on *spr*, consistent with a model in which NlpI controls the activity of the PG endopeptidase Spr. In log phase, the *nlpI* mutation was suppressed by a *dacB* mutation, suggesting that NlpI regulates penicillin-binding protein 4 (PBP4) during exponential growth. The data support a model in which NlpI negatively regulates PBP4 activity during log phase, and Spr activity during stationary phase, and that in the absence of NlpI, the cell survives by increasing PG synthesis. Further, the *nlpI* mutant exhibited a significant decrease in covalent outer membrane (OM-PG) envelope stabilizing cross-links, consistent with its high level of OMV production. Based on these results, we propose that one mechanism wild-type Gram-negative bacteria can use to modulate vesiculation is by altering PG-OM cross-linking via localized modulation of PG degradation and synthesis.

## Introduction

All Gram-negative bacteria studied to date ubiquitously secrete outer membrane vesicles (OMVs), which are spherical heterogeneous structures on the order of 50–250 nm, emanating from the outer membrane (OM) containing an OM exterior and periplasmic lumenal content from the site of budding (Beveridge 1999; Kulp and Kuehn 2010; Deatherage and Cookson 2012; Berleman and Auer 2013; Schwechheimer et al. 2013). OMVs serve in numerous functional roles to mediate interactions of Gram-negative bacteria with their environment (Schooling and Beveridge 2006; Yonezawa et al. 2009; Ellis and Kuehn 2010; Deatherage and Cookson 2012; MacDonald and Kuehn 2013a). Their functions have been elucidated primarily in the areas of host–pathogen interactions and multispecies environments such as biofilms, but they also benefit individual bacteria as a stress response, protecting the originating cell from harmful internal or external agents (McBroom and Kuehn 2007; Tashiro et al. 2009; Manning and Kuehn 2011; Maredia et al. 2012; McMahon et al. 2012; MacDonald and Kuehn 2013b; Schwechheimer and Kuehn 2013).

Within the periplasmic space, sandwiched between the cytoplasmic membrane and the OM, the peptidoglycan (PG, or murein) forms a net-like structure composed of glycan chains cross-linked by short peptides (Silhavy et al. 2010). The PG encapsulates the hypertonic cytoplasm and protects Gram-negative cells from lysis due to osmotic changes and mechanical stress (Vollmer and Bertsche 2008). For envelope stability, the PG is covalently cross-linked to the OM via the short OM-anchored lipoprotein Lpp (Braun and Rehn 1969; Braun and Wolff 1975). A deletion of *lpp* results in a fragile envelope structure associated with membrane shedding and cellular leakage (Braun and Wolff 1975; Cascales et al. 2002; Deatherage et al. 2009).

We propose that by analyzing mutants with OM vesiculation phenotypes that do not have compromised membrane integrity, we can begin to elucidate the mechanistic basis of OMV production. Previously, our laboratory screened for and identified numerous transposon insertions with altered vesiculation phenotypes in *Escherichia coli* (McBroom et al. 2006). One particularly strong hypervesiculating mutant contained an insertion at the beginning of the *nlpI* gene. The *nlpI*-encoded gene product, NlpI, is an OM-anchored lipoprotein that has been associated with cell division but its precise function has not yet been determined (Ohara et al. 1999; Pierce et al. 2011). Notably, a mutation in *nlpI* was found to suppress temperature sensitivity associated with a mutation in *spr* which encodes a murein DD-endopeptidase (Tadokoro et al. 2004; Singh et al. 2012). Spr is most likely also an OM lipoprotein based on sequence structure, although its lipidation has not been experimentally demonstrated (Uniprot 2015).

Phenotypic screening also revealed mutants with hypovesiculation defects. Individual deletions of *nlpA*, *dsbA*, and *bolA* produce fewer OMVs than wild type (WT), and further study showed that each deletion partially suppressed another hypervesiculating mutant, Δ*degP* (Schwechheimer and Kuehn 2013). DegP is a periplasmic chaperone and protease that manages misfolded periplasmic protein stress (Spiess et al. 1999; Raivio and Silhavy 2001; Ortega et al. 2009). In the Δ*degP* mutant, misfolded proteins that accumulate are shed via OMVs, and the ability of this strain to hypervesiculate prevents the otherwise negative effects of toxic periplasmic protein accumulation on bacterial growth (Schwechheimer and Kuehn 2013). Why mutations in *nlpA*, *dsbA*, and *bolA* reduce vesiculation levels is not yet understood: NlpA, DsbA, and BolA play a role in methionine import, periplasmic disulfide bond formation, and stationary phase transition, respectively (Kamitani et al. 1992; Bardwell et al. 1993; Zhang et al. 2003; Freire et al. 2009, 2006; Schwechheimer and Kuehn 2013).

In this study, we aimed to gain insight into envelope control of OMV production by analyzing the role of *nlpI*. We analyzed and compared OMV production in single mutants and mutants containing multiple mutations. Since the genetic interaction of *nlpI* and *spr* implicated the involvement of PG metabolism in the phenotype, we analyzed the sensitivity of strains to a PG synthesis inhibitor. In addition, we compared the quantity of covalent Lpp-OM cross-links to establish a potential mechanistic basis for differences in OMV production in the strains. Together, the data support a model in which NlpI negatively regulates PG endopeptidase activity, and that in the absence of NlpI, the cell survives by increasing PG synthesis. Furthermore, the increase in PG dynamics proposed to occur in the *nlpI* mutant correlates with an observed decrease in Lpp-OM cross-linking, which likely contributes to the hypervesiculation phenotype.

## Materials and Methods

### Growth conditions and reagents

Strains and plasmids used in this work are summarized in Tables1 and S2. Bacteria were grown at 37°C in liquid culture in EM Science Luria–Bertani (LB) broth, Miller, pH 7.2 (EMD Millipore, Billerica, MA, USA) at 25°C or on plates of solid LB agar supplemented with 50 mg/mL kanamycin or 100 mg/mL ampicillin (Sigma-Aldrich, St. Louis, MO USA). Isopropyl *β*-d-1-thiogalactopyranoside (IPTG) (VWR International, Atlanta, GA, USA) was added to induce protein expression if indicated. The single gene mutants originate from the Keio Collection (Baba et al. 2006). To create mutants with multiple deletions, the kanamycin resistance marker was removed from the single mutant (Cherepanov and Wackernagel 1995). The additional mutation was then added by transduction of the marked gene deletion using P1 phage (Silhavy et al. 1984) from the donor single Keio mutant strain into the unmarked Keio recipient mutant strain. The mutants constructed for this work were either sequenced with a primer upstream and downstream of the deleted gene or PCR amplified with primers upstream/downstream of the deleted gene and the kanamycin cassette to confirm the genotypes.

**Table 1:** Strains and plasmids

Strains	Genotype	Source/reference
*Escherichia coli*		
BW25113	*rrnB3 ΔlacZ4787 hsdR514 Δ(araBAD)567Δ(rhaBAD)568 rph-1*	WT of Keio collection Baba et al. (2006)
Keio collection single mutants	BW25113 with indicated single mutations: Δ*nlpA*::Kan, Δ*degP*::Kan, Δ*dsbA*::Kan, Δ*bolA*::Kan, Δ*nlpI*::Kan, Δ*spr*::Kan, Δ*lpp*::Kan, Δ*pbpG*::Kan	Baba et al. (2006)
MK1277	BW25113 Δ*ycfS*, Δ*ybiS*, Δ*erfK*::*Kan*	Schwechheimer et al. (2013)
MK1280	BW25113 Δ*nlpI*, Δ*spr*::*Kan*	This work
MK1281	BW25113 Δ*nlpI*, Δ*nlpA*::*Kan*	This work
MK1282	BW25113 Δ*nlpI*, Δ*dsbA*::*Kan*	This work
MK1283	BW25113 Δ*nlpI*, Δ*bolA*::*Kan*	This work
MK1284	BW25113 Δ*nlpI*, Δ*nlpC*::*Kan*	This work
MK1285	BW25113 Δ*nlpI*, Δ*ydhO*::*Kan*	This work
MK1286	BW25113 Δ*nlpI*, Δ*yafL*::*Kan*	This work
MK1287	BW25113 Δ*nlpI*, Δ*yebA*::*Kan*	This work
MK1288	BW25113 Δ*spr*, Δ*degP*::*Kan*	This work
MK1289	BW25113 Δ*nlpI*, Δ*spr*, Δ*degP*::*Kan*	This work
MK1340	BW25113 Δ*nlpI*, Δ*pbpG*::*Kan*	This work
MK1341	BW25113 Δ*nlpI*, Δ*mepA*::*Kan*	This work
MK1342	BW25113 Δ*nlpI*, Δ*dacB*::*Kan*	This work
MK1263	BW25113/pTrc99A	Schwechheimer and Kuehn (2013)
MK1290	BW25113 Δ*nlpI*::*Kan*/pTrc99A	This work
MK1291	BW25113 Δ*nlpI*::*Kan*/pNlpI	This work
MK1292	BW25113 Δ*nlpI*, Δ*spr*::*Kan*/pTrc99A	This work
MK1293	BW25113 Δ*nlpI*, Δ*spr*::*Kan*/pNlpI	This work
MK1294	BW25113 Δ*nlpI*, Δ*spr*::*Kan*/pSpr	This work
MK1296	BW25113 Δ*spr*::*Kan*/pTrc99A	This work
MK1297	BW25113 Δ*spr*::*Kan*/pSpr	This work
MK1299	BW25113 Δ*spr*::*Kan*/pmSpr	This work
Plasmids		
pTrc99A	trc promoter, Amp^R^, pBR322 ori	
pNlpI	*nlpI* in pTrc99A, Amp^R^, IPTG-inducible	This work
pSpr	*spr* in pTrc99A, Amp^R^, IPTG-inducible	This work
pmSpr	pSpr with C68A point mutation	This work

WT, wild-type; IPTG, isopropyl *β*-d-1-thiogalactopyranoside

For the d-Met stationary phase growth curves, cultures (25 mL) were inoculated (1:250 dilution) and grown overnight (∽16 h) at 37°C. Cells were pelleted with an Beckman Avanti J-25 centrifuge (JLA-16.250 rotor, 10,000*g*, 10 min, 4°C), resuspended in fresh media (25 mL) treated with the indicated concentration of d-Met or the same volume of LB (untreated controls), and incubated (6 h, 37°C) with hourly optical density measurements at 600 nm (OD_600_).

### Construction of expression vectors

In order to express *nlpI* and *spr* under control of the trc promoter, pNlpI and pSpr were constructed using the pTrc99A vector. *nlpI* was amplified from genomic DNA using the primers 5′-GATATCTAGAGCCCTCCGCTGCGGC-3′ containing the XbaI restriction site and 5′-GATTAAGCTTCTATTGCTGGTCCGATTCTGCA-3′ containing the HindIII restriction site. The *nlpI*-containing XbaI-HindIII fragment was then ligated with the XbaI-HindIII cleaved pTrc99A fragment. *spr* was amplified from genomic DNA using the primers 5′-AAAAGGATCCGTCTCGTGTCGCTTGGC-3′ containing the BamHI restriction site and 5′-AAAACTGCAGCTCGTCAGGATAGCCAAGG-3′ containing the PstI restriction site. The *spr*-containing BamHI-PstI fragment was then ligated with the BamHI-PstI cleaved pTrc99A fragment. The restriction enzymes were purchased from New England Bio Labs (Ipswich, MA, USA). A pSpr derivative, pmSpr, was created to express mutant Spr using the inducible *trc* promoter. The point mutation was introduced by site directed mutagenesis of pSpr using the primers 5′-GGTATCGATGCGTCTGGTTTCGTACAGCG-3′ and 5′-CGCTGTACGAAACCAGACGCATCGATACC-3′. Plasmids expressing C-terminally FLAG-tagged versions of Spr and mSpr (pSpr-F and pmSpr-F, respectively) were made from the Spr and mSpr constructs in pTrc99A using the same forward primer as above 5′-AAAAGGATCCGTCTCGTGTCGCTTGGC-3′ and the reverse primer containing the FLAG-tag 5′-AACTGCAGTCATTTGTCATCGTCATCTTTATAATCTCTAGAGCTGCGGCTGAGAACCCG-3′. The plasmid constructions were confirmed by sequencing using a primer upstream and downstream of the cloned gene.

### OMV purification and quantitation

Unless indicated, media (250 mL) was inoculated (1:250 dilution) from bacterial cultures grown overnight at 37°C and cells were grown overnight again at 37°C (∽16 h). Cells were pelleted with the Beckman Avanti J-25 centrifuge (JLA-10.500 rotor, 10,000*g*, 10 min, 4°C) and the resulting supernatants filtered (low protein binding Durapore membrane, 0.45 *μ*m polyvinylidene fluoride, Millipore, Billerica, MA, USA). Filtrates were centrifuged again with the Beckman Avanti J-25 centrifuge (JLA-16.250 rotor, 38,400*g*, 3 h, 4°C) followed by another step of centrifugation with the Beckman Optima TLX Ultracentrifuge (Beckman Coulter, Inc., Indianapolis, IN, USA) if the pellets were not visible. In these cases, most of the supernatant was poured off, and the region where pelleted material should be was “resuspended” in the residual supernatant and re-pelleted (TLA 100.3 rotor, 41,000*g*, 1 h, 4°C). Pellets were resuspended in Dulbecco's phosphate-buffered saline with added salt (0.2 mol/L NaCl), and filter-sterilized through 0.45 *μ*m Ultra-free spin filters (Millipore). A portion of the filtrate was plated on LB agar and incubated at 37°C overnight to verify that the suspensions were free of bacteria.

To quantitate OMV yield, OMV preparations were boiled for 6 min in 2X Laemelli buffer, separated by 15% sodiumdodecyl sulphate-polyacrylamide gel electrophoresis (SDS-PAGE), and stained with SYPRO Ruby Red (Molecular Probes, Grand Island, NY, USA) overnight in the dark. Prior to and after staining, the gel was fixed for 1 h in a solution of 10% MeOH and 7% acetic acid. Ruby-stained proteins were detected under UV light. *E. coli* Omps F/C and A were quantified by densitometry (NIH Image J software, National Institutes of Health, Bethesda, MD, USA). The Outer membrane protein (Omp) density values were divided by the OD_600_ of the original culture to calculate OMV production and this value was divided by the OMV production of the WT strain or untreated control strain to determine relative fold OMV production.

### PG purification, digestion, and quantitation of covalently cross-linked Lpp

Unless otherwise indicated, media (500 mL) was inoculated (1:250 dilution) from overnight 37°C bacterial cultures and cultures grown at 37°C until they reached OD_600_ ∽0.4. Sacculi were isolated from broth cultures based on the protocol by Lam et al. (2009). Briefly, cells were pelleted and resuspended in PBS after which the ice-cold suspensions were dropped in an equal volume of vigorously stirring, boiling 10% SDS. Samples were boiled for 4 h and then incubated at 37°C, continuously shaking, overnight. The following day, the PG was pelleted with the Beckman Optima TLX Ultracentrifuge (TLA 100.3 rotor, 80,000*g*, 15 min, 30°C), resuspended in 1% SDS followed by another 2 h of boiling. PG was washed four times with deionized water and finally resuspended in equal volumes of deionized water.

Equal fractions of the purified sacculi were digested with 15 mg/mL chicken egg lysozyme (Sigma-Aldrich, St. Louis, MO, USA) in 10 mmol/L Tris-HCL, pH 8, at room temperature for 2 days. Lysozyme digested PG was separated by 15% SDS-PAGE and Lpp was detected by immunoblotting and quantified by densitometry (NIH Image J software). The Lpp density values were divided by the OD_600_ of the original culture to calculate the amount of Lpp that was covalently cross-linked to PG, and this value was divided by the PG-cross-linked Lpp of the WT strain or untreated control strain to determine relative fold of bound Lpp.

The concentration of free Lpp in the samples was determined by boiling whole-cell samples in Laemelli buffer and 1% SDS in PBS without lysozyme prior to SDS-PAGE (Cowles et al. 2011). Lpp was detected by immunoblotting using an anti-Lpp antibody (Cowles et al. 2011; generously provided by the Silhavy laboratory) and quantified by densitometry (NIH Image J software). The density values of the Lpp band were divided by the OD_600_ of the original culture to calculate the amount of free Lpp, and this value was divided by the Lpp of the WT strain to determine relative fold of free Lpp. We decided to normalize bound Lpp to OD_600_ rather than then the traditional method (ratio of bound Lpp to total PG), since this provides a better measure of the number of OM-PG linkages per cell rather than the number of OM-PG linkages per amount of PG (which could vary in thickness). Therefore, this value can reflect how readily the OM is able to bud off as an OMV.

### Statistics

Parameters used for the *T*-test are equal variance due to the comparison of identical experimental repetitions or unequal variance due to different experimental repetitions and a two-tail distribution. For direct sample size comparison, the paired *T*-test was used, and for fold comparison, the unpaired. The *T*-test value of ≤0.05 was considered statistically significant; if the value was lower than 0.05, the significance value is given under the corresponding data. The number of times each experiment was repeated (*n*) is stated in the figure legends.

## Results

### A deletion in *spr* specifically suppresses the hypervesiculation phenotype of the Δ*nlpI* mutant

**Figure 1:**
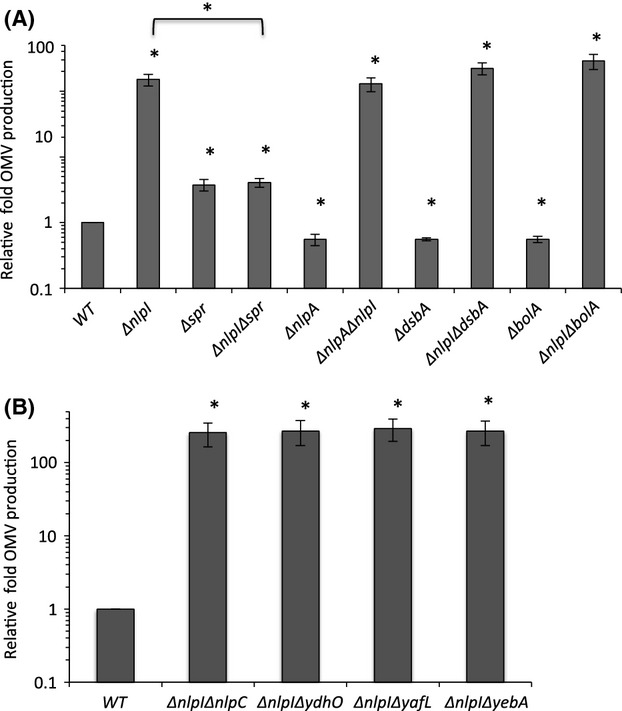
Deletion of *spr* specifically suppresses the hypervesiculation phenotype of the Δ*nlpI* mutant. (A and B) Relative fold OMV production in cultures of the indicated strains grown in LB overnight at 37°C was determined by quantitating OMVs, normalizing to OD_600_, and dividing by OD_600_-normalized OMV production in a WT culture. Statistical comparisons are with WT levels unless denoted by a bracket. *P ≤ 0.05; *n* ≥ 3. Error bars indicate standard error of the mean (SEM). OMV, outer membrane vesicles; LB, Luria–Bertani; WT, wild-type.

First, we ensured that, like the N-terminal transposon insertion mutant discovered earlier, the *E. coli* full deletion mutant of *nlpI* (Δ*nlpI*) hypervesiculated (Fig.1A) and could be complemented with a high-copy expression plasmid (pTrc99A) carrying *nlpI* (pNlpI). OMVs were purified and quantified via previously established methods (McBroom et al. 2006). Uninduced expression of NlpI was sufficient to nearly completely reduce OMV production to the amount produced by the isogenic WT strain (Fig. S1). It should be noted that all strains and treatments assayed in this study were assessed for growth and membrane integrity, as described previously (Schwechheimer and Kuehn 2013) so that we could be confident that changes in OMV production levels were not due to substantial differences in cell density or lysis. These data are summarized in Table S1 and included in full in the Data S1.

A genetic association between *nlpI* and *spr* had been reported previously (Tadokoro et al. 2004), therefore we investigated whether the loss of *spr* had an effect on the strong hypervesiculation phenotype of the Δ*nlpI* mutant. We found a modest hypervesiculation phenotype for the Δ*spr* mutant and a similar modest phenotype for the Δ*nlpI*Δ*spr* double mutant (Fig.1A). Therefore, the deletion of *spr* reduced OMV production by the Δ*nlpI* strain ∽30-fold.

To examine the specificity of the *spr* deletion on the suppression of the Δ*nlpI*-associated hypervesiculation phenotype, deletion mutations in *nlpA*, *dsbA*, and *bolA* were introduced into the Δ*nlpI* strain. Unlike their ability to partially suppress another hypervesiculating mutant, Δ*degP* (Schwechheimer and Kuehn 2013), and unlike the deletion in *spr*, none of these deletions had an effect on the hypervesiculation phenotype of the Δ*nlpI* mutant (Fig.1A). To further examine the specificity of the interaction of *spr* and *nlpI*, we determined if deletions in genes encoding the three structural and/or functional Spr homologs, *ydhO*, *yafL*, and *yebA* (Singh et al. 2012), could also suppress the hypervesiculation phenotype of the Δ*nlpI* mutation. Among this family, only the *spr* deletion reduced OMV production of the Δ*nlpI* mutant (Fig.1A and B).

Suppression of the thermosensitive growth of *spr* mutants could be achieved, not only by the loss of *nlpI*, but also by the overexpression of penicillin-binding protein (PBP) 7, another murein DD-endopeptidase which is encoded by *pbpG* (Romeis and Holtje 1994; Tadokoro et al. 2004). These phenotypes suggested that NlpI may play a role in the negative regulation of PBP7 activity. However, when we examined whether the deletion of *pbpG* would reduce OMV production of the Δ*nlpI* mutant, we found that this was not the case (Fig. S2A). Together, these data demonstrate the specific role in OMV production that Spr plays with respect to NlpI.

### Hypervesiculation by Δ*degP* is independent of the *nlpI*/*spr* pathway

**Figure 2:**
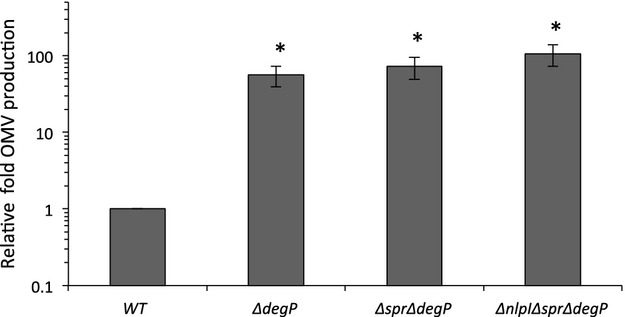
Deletion of *degP* causes hypervesiculation in the Δ*nlpI*Δ*spr* mutant. Relative fold OMV production in cultures of the indicated strains was determined as in Figure1. Statistical comparisons are with WT levels. *P ≤ 0.05; WT, *n* ≥ 5. Error bars indicate SEM. OMV, outer membrane vesicles; WT, wild-type.

We next investigated the converse question: whether the deletion of *spr* reduced OMV production for any other hypervesiculating mutant. We examined OMV production for the Δ*spr*Δ*degP* double mutant and found that the loss of *spr* had no effect on hypervesiculation caused by the deletion of *degP* (Fig.2), further supporting the specific relationship between *nlpI* and *spr*.

In order to understand the specificity of the Δ*nlpI*Δ*spr* mutant phenotype, we also needed to consider whether the Δ*nlpI*Δ*spr* double mutation simply prevented the ability to overproduce OMVs, perhaps due to the loss of some critical functional component. To address this, we tested whether the hypervesiculation phenotype of the Δ*degP* mutant could be suppressed by the Δ*nlpI*Δ*spr* double mutation. The data demonstrate that the triple mutant could, in fact, hypervesiculate (Fig.2), and was consistent with the inability of mutations in *nlpA*, *dsbA*, and *bolA* to affect the Δ*nlpI* phenotype (Fig1A). Together, these results revealed that *spr* and *nlpI* function in an OMV production pathway that is independent from the pathway leading to hypervesiculation by Δ*degP*.

### Δ*nlpI* is sensitive to an inhibitor of PG synthesis and sensitivity is specifically suppressed by Δ*spr*

Since Spr had recently been identified as a PG DD-endopeptidase (Singh et al. 2012), and the loss of *spr* specifically suppressed hypervesiculation associated with the Δ*nlpI* mutation, we hypothesized that the increased OMV production of the Δ*nlpI* mutant may result from an increase in PG degradation by uncontrolled Spr activity in the mutant. Since PG is essential (Weidel and Pelzer 1964; Vollmer and Bertsche 2008), it follows that the continuous PG degradation by overactive Spr would be counteracted by upregulation of PG synthesis. Thus, in the absence of NlpI, continuous synthesis of new PG, even in stationary phase, is likely necessary to allow the cell to survive uncontrolled Spr-mediated PG degradation.

**Figure 3:**
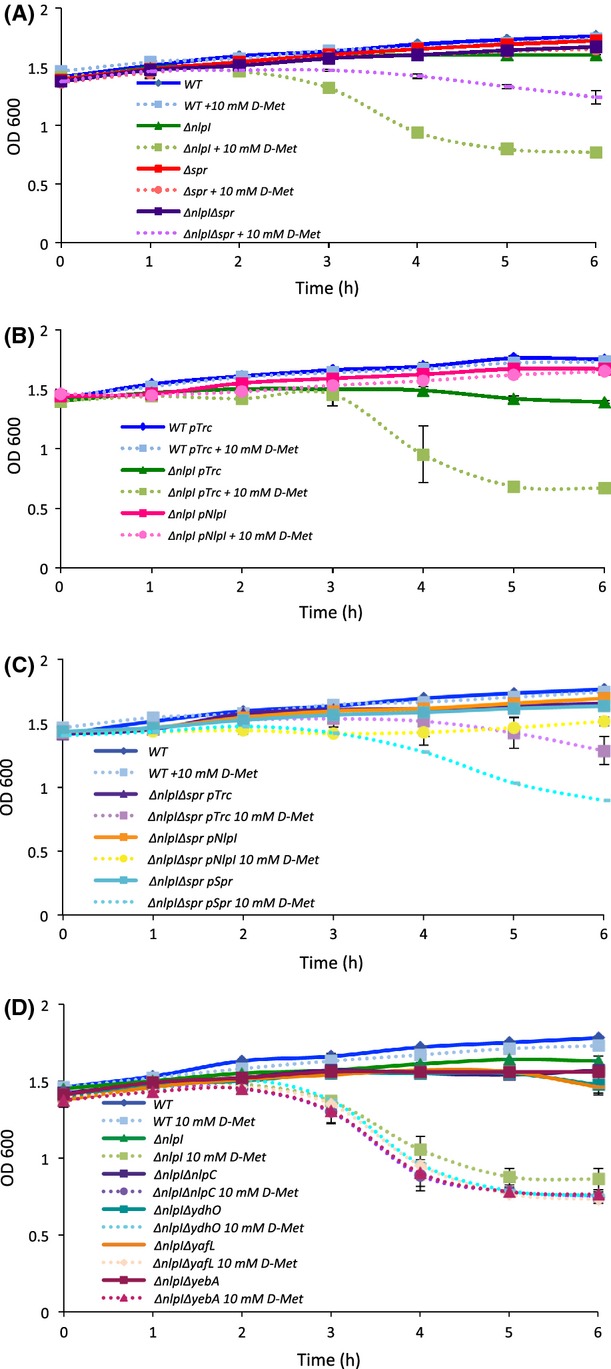
Sensitivity to d-Met during nonexponential phase growth indicates high PG turnover in the Δ*nlpI* mutant. (A and D) Cultures of the indicated strains were grown in LB overnight at 37°C, cells were pelleted and resuspended into fresh LB (*t* = 0) ±10 mmol/L d-Met, and then grown at 37°C. OD_600_ was measured hourly. Data represent the average of two independent experiments. (B and C) Cultures of the indicated strains carrying the vector (pTrc), or NlpI- or Spr-encoding plasmids, were grown in LB overnight at 37°C, cells were pelleted and resuspended into fresh LB (*t* = 0) ±10 mmol/L d-Met, and then grown at 37°C. OD_600_ was measured hourly. Data represent the average of two independent experiments. LB, Luria–Bertani; PG, peptidoglycan.

If PG synthesis were upregulated in the Δ*nlpI* mutant, we anticipated that this would be particularly evident in stationary phase, when PG synthesis is typically negligible in a WT cell. To investigate this hypothesis, we compared the sensitivity of stationary phase cultures to treatment with 10 mmol/L d-methionine (d-Met), which has an inhibitory effect on PG synthesis (Caparros et al. 1992; Lam et al. 2009). In “pseudo-stationary phase” cultures (for this analysis, the cultures had to be resuspended in fresh media, therefore it was not termed “stationary phase”), 10 mmol/L d-Met would be expected to have minimal effects on the WT strain because of the low rate of PG synthesis, whereas mutants with increased PG degradation would require increased PG synthesis and consequently would be sensitive and exhibit a decrease in culture density. We assessed the sensitivity of pseudo-stationary phase cultures of the mutant and WT strains to treatment with 10 mmol/L d-Met by monitoring OD_600_. As suspected, the *nlpI* deletion strain was hypersensitive to 10 mmol/L d-Met: after 2 h, the OD_600_ dropped off dramatically, whereas the WT strain was unaffected (Fig.3A). To ensure that it was indeed the loss of *nlpI* that caused d-Met hypersensitivity, we complemented the deletion strain with pNlpI and found that uninduced expression of NlpI was sufficient to reduce d-Met sensitivity of the Δ*nlpI* mutant to a WT level (Fig.3B). These data suggest that the Δ*nlpI* mutant actively synthesizes PG even in pseudo-stationary phase. The Δ*nlpI*Δ*spr* mutant showed only a modest decrease in OD_600_ with 10 mmol/L d-Met, indicating that the double mutant strain is less active for PG synthesis and more similar to the WT strain (Fig.3A). The suppression of Δ*nlpI-*dependent d-Met sensitivity by a deletion in *spr* supports our hypothesis that NlpI negatively regulates Spr activity.

To examine the d-Met hypersensitivity phenotype further, we analyzed growth of the Δ*nlpI*Δ*spr* mutant transformed with either pSpr (*spr* in the pTrc99A high-copy expression plasmid) or pNlpI. As expected, expression of Spr increased d-Met hypersensitivity of the double mutant (Fig.3C), displaying a phenotype similar to that of the Δ*nlpI* strain (Fig.3A), whereas expression of NlpI did not have much of an effect on d-Met hypersensitivity (Fig.3C), displaying a phenotype similar to that of the Δ*spr* strain (Fig2A). These data support the hypothesis that unregulated Spr activity can cause sensitivity to d-Met in pseudo*-*stationary phase.

To again address the question of specificity, we investigated whether any of Spr's structural or functional homologs could suppress the d-Met sensitivity of the Δ*nlpI* mutant. Consistent with the lack of suppression seen for the hypervesiculation phenotype in Figure1B, none of the *spr* homolog deletions could suppress d-Met hypersensitivity of the Δ*nlpI* mutant (Fig.3D). We also investigated if Δ*pbpG* could suppress d-Met hypersensitivity of the Δ*nlpI* mutant. Again, as seen for OMV production (Fig. S2A), no improvement in growth with d-Met was observed for the Δ*nlpI*Δ*pbpG* double mutant (Fig. S2B). Together, the d-Met sensitivity data (Figs.3, and S2B) show that the Δ*nlpI* mutant exhibits *spr*-dependent elevated levels of PG synthesis in pseudo-stationary phase. The results are consistent with our hypothesis that cells lacking *nlpI* increase PG synthesis to compensate for increased PG degradation by unregulated Spr activity.

### Loss of *dacB*, not *spr,* rescues d-Met hypersensitivity of the Δ*nlpI* mutant in log phase

**Figure 4:**
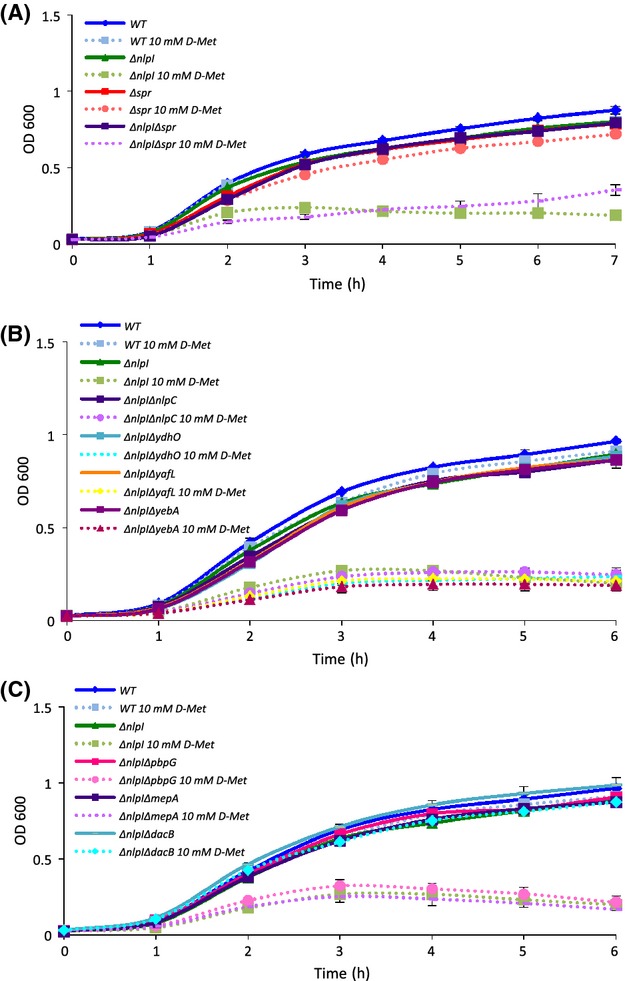
In log phase, deletion of *dacB* specifically suppresses d-Met sensitivity of the Δ*nlpI* strain. (A–C) Indicated strains of bacteria were grown in LB overnight at 37°C, inoculated into fresh LB (*t* = 0), and then grown at 37°C. OD_600_ was measured hourly. Data represent the average of two independent experiments. LB, Luria–Bertani.

Spr activity is most critical during active growth and cell division in log phase (Singh et al. 2012), thus we hypothesized that Spr activity suppression by NlpI would be decreased or even nonexistent during log phase, when maximum Spr activity is required by the cell. To test this, we first examined whether the Δ*nlpI* mutant was sensitive to d-Met in log phase and found that this was the case (Fig.4A). However, under these conditions, the additional deletion of *spr* did not provide rescue (Fig.4A). Similarly, none of the *spr* homolog mutants reduced the *nlpI* deletion d-Met sensitivity (Fig.4B). These data suggested that NlpI may negatively regulate another PG hydrolase.

As mentioned previously, it had been noted that *spr* mutants could be rescued by additionally mutating *nlpI* (Tadokoro et al. 2004), indicating that the relief came from increased activity of an enzyme with a similar function as Spr that is also negatively regulated by NlpI. To test whether we could identify the gene responsible for this activity, we constructed double deletion mutants of *nlpI* with each of *E. coli's* additional three endopeptidases: *pbpG*, *mepA,* or *dacB*. We found that the loss of *dacB*, the gene that encodes PBP4, suppressed d-Met hypersensitivity of the Δ*nlpI* mutant in log phase (Fig.4C). These data suggest that NlpI can negatively regulate PBP4 activity during active growth. Furthermore, it provides a plausible explanation why the thermosensitivity associated with an *spr* mutation could be rescued by the overexpression of Pbp7 (Hara et al. 1996), a functional homolog of Pbp4 (Vollmer and Bertsche 2008): If NlpI inhibition of Pbp4 is increased in an *spr* mutant, then since Pbp7 is also a PG hydrolase, overexpression of this homolog could provide relief.

### Induced expression of functional Spr results in d-Met sensitivity and hypervesiculation

**Figure 5:**
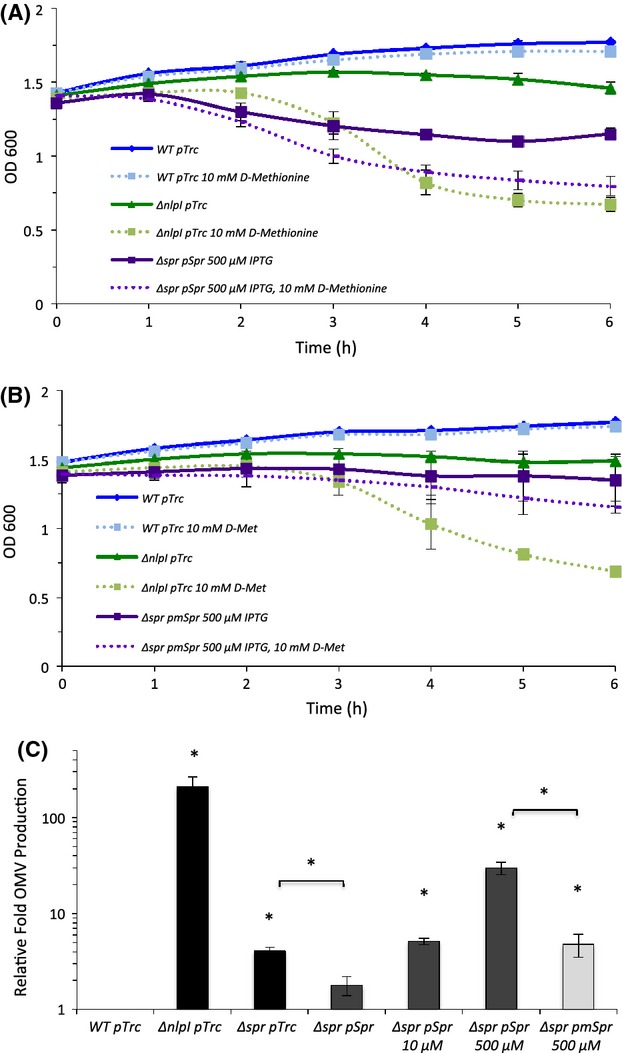
Similar d-Met sensitivity and OMV phenotypes result from Spr overexpression and the deletion of *nlpI*. (A and B) Indicated strains of bacteria carrying the vector, or plasmids encoding NlpI, Spr, or mSpr, were grown in LB overnight at 37°C (induced at inoculation if indicated), cells were pelleted and resuspended into fresh LB (*t* = 0) ±10 mmol/L d-Met, and then grown at 37°C. OD_600_ was measured hourly. Data represent the average of two independent experiments. (C) Relative fold OMV production in cultures of the indicated strains grown in LB overnight at 37°C (induced at inoculation if indicated) was determined as described in Figure1. *P* values refer to comparisons with WT unless denoted by a bracket. *P ≤ 0.05; *n* ≥ 3. Error bars indicate SEM. OMV, outer membrane vesicles; LB, Luria–Bertani; WT, wild-type.

If NlpI acts as a negative regulator of Spr late in the bacterial life cycle, then overexpression of Spr should yield similar phenotypes as the deletion of *nlpI*. To test this, we analyzed stationary phase cultures of a highly induced (500 *μ*mol/L IPTG) Δ*spr* pSpr strain. Even without the addition of d-Met, strong induction of Spr lead to a reduction in the culture density with respect to the WT strain, mimicking the sensitivity of the Δ*nlpI* mutant (Fig.5A). As expected, the addition of 10 mmol/L d-Met had a further detrimental effect on cells overexpressing Spr (Fig.5A). These results were consistent with the d-Met sensitivity observed for the Δ*nlpI*Δs*pr* double mutant complemented with pSpr (Fig.3C).

We next wanted to establish whether it was the PG endopeptidase activity of Spr that caused these phenotypes. We constructed an expression plasmid encoding Spr (pmSpr) with a point mutation in the catalytic triad (Cys68 changed to Ala, C68A), which has been previously shown to cause impaired PG endopeptidase activity (Aramini et al. 2008; Singh et al. 2012). To ensure that the mutation did not interfere with expression and OM localization, we constructed a C-terminal FLAG-tagged version of Spr (pSpr-F) and mutant Spr (pmSpr-F) in order to be able to monitor Spr and mSpr location and expression levels. The FLAG-tag did not interfere with Spr function, since basal and induced expression (with 500 *μ*mol/L IPTG) of Δ*spr* pSpr-F yielded results very similar to those using the untagged version (Figs. S3 and 5C), although OMV production was observed to be slightly higher. Both pSpr-F and pmSpr-F localized to the OM and were expressed similarly (Fig. S4), indicating that the C68A mutation did not disturb localization or stability. Therefore, any phenotypes observed for the mutant would be due to a result of the loss of activity, rather than a decrease in expression or mislocalization. When we compared the effect of 10 mmol/L d-Met treatment of strains expressing mSpr and Spr, we observed that increased expression of mSpr resulted in a moderate growth defect, but nowhere near as detrimental as the overexpression of functional Spr (Fig.5B), supporting the hypothesis that it is the endopeptidase activity of Spr that is negatively regulated by NlpI.

Next, we examined if overexpression of the functional form of Spr could significantly increase OMV production compared to overexpression of the mutant Spr. A slight elevation in OMV levels was also expected for the mutant, since we have shown previously that increased envelope protein expression in general can lead to hypervesiculation (McBroom and Kuehn 2007; Schwechheimer and Kuehn 2013). We found that uninduced expression of functional Spr in the Δ*spr* background complemented the slight OMV overproduction phenotype of the Δ*spr* strain to a WT level, as expected, whereas vesiculation increased in an IPTG concentration-dependent manner (Fig.5C). It is noticeable that vesiculation driven by induced Spr does not reach the level of the *nlpI* mutant. We suspect this is due to the sensitivity to nonnative timing of Spr expression for the plasmid-expressed gene. When the experiment was repeated with mSpr induced with 500 *μ*mol/L IPTG, we found that OMV production was slightly elevated with respect to WT levels (as anticipated), but significantly less than functional Spr. In summary, these data strengthen the hypothesis that NlpI negatively and specifically regulates the DD-endopeptidase activity of Spr, impacting OMV production.

### PG-cross-linked Lpp levels negatively correlate with OMV production levels in the Δ*nlpI and* Δ*nlpI*Δ*spr* mutants

Our discovery of the relationship between OMV production and PG turnover led us to consider the effect of PG turnover on the number of covalent crosslinks between the OM and PG and the potential downstream effect on OMV production. In the case of the Δ*nlpI* mutant, the proposed high rate of PG synthesis, might lead to low numbers of crosslinks, causing a loosely associated OM and consequent hypervesiculation.

**Figure 6:**
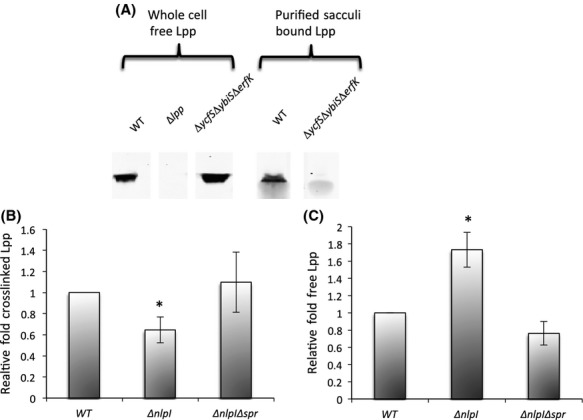
PG cross-linked Lpp inversely correlates with OMV production in the Δ*nlpI* mutant, and levels are restored with *spr* deletion. (A) Anti-Lpp immunoblot of whole cell and copurified PG of the indicted strains. Composite figure created from the rearrangement of lanes from a single immunoblot. (B) Relative fold cross-linked Lpp in cultures of the indicated strains grown in LB to an OD_600_ of ∽0.4 at 37°C was determined by quantitative immunoblotting of Lpp copurified with PG, normalizing to OD_600_, and dividing by OD_600_-normalized cross-linked Lpp in a WT culture. (C) Relative fold of free Lpp in cultures of the indicated strains grown overnight in LB. *P* value refers to comparison with WT. *P ≤ 0.05; *n* > 3. Error bars indicate SEM. OMV, outer membrane vesicles; LB, Luria–Bertani; WT, wild-type; PG, peptidoglycan.

To investigate this hypothesis, we developed an immunoblotting assay that takes advantage of the fact that Lpp isolated with purified PG sacculi is solely in its cross-linked form. This form has been historically referred to as the “bound” form due to its covalent linkage to PG. The “free” form of Lpp is considered to be the form that is OM-anchored via the protein's lipid moiety, but not covalently cross-linked to PG. After sacculi isolation, lysozyme digestion, and SDS-PAGE, bound Lpp was quantified by immunoblotting. Whole-cell extract of the *lpp* deletion strain, Δ*lpp*, was used as a negative control for immunoblotting (Fig.6A). To establish that we could distinguish between the bound and free forms of Lpp, we utilized a triple mutant strain, Δ*ycfS*Δ*ybiS*Δ*erfK*, that expresses only free Lpp because it lacks the enzymes that form the covalent bond (Magnet et al. 2007). We first verified that similar levels of free Lpp were present in whole-cell preparations of the WT strain and the triple mutant strain (Fig.6A). The concentration of free Lpp in the samples was determined by boiling whole-cell samples in Laemelli buffer and 1% SDS in PBS without lysozyme prior to SDS-PAGE (Cowles et al. 2011). Next, we isolated and analyzed purified PG sacculi from these strains. Lpp could be detected in PG sacculi from the WT strain, whereas PG sacculi from the triple mutant contained no detectable Lpp (Fig.6A). These data showed that this assay allows selective quantitation of bound Lpp.

The levels of covalent Lpp cross-linking in the WT, Δ*nlpI,* and Δ*nlpI*Δ*spr* strains were compared using our bound Lpp assay. We found significantly reduced levels (∽40%) of covalently cross-linked Lpp for the Δ*nlpI* strain with respect to WT levels, however, we found WT levels of cross-linked Lpp for the Δ*nlpI*Δ*spr* double mutant (Fig.6B). To establish that the observed decrease of bound Lpp in the Δ*nlpI* mutant was not a reflection of overall decreased Lpp expression, we evaluated whether there was a corresponding increase in the level of free Lpp in this strain. We found that, indeed, levels of free Lpp in the Δ*nlpI* strain were higher than in the WT strain (Fig.6C). To ensure that the deletion in *nlpI* did not affect Lpp localization, we examined whether Lpp fractionated with the PG in the Δ*ycfS*Δ*ybiS*Δ*erfK*Δ*nlpI* mutant. Lpp fractionated similarly for this mutant and the triple mutant expressing only free Lpp (Compare Figs.6A and S5A). These data support our hypothesis that NlpI regulation of Spr-dependent PG hydrolysis affects Lpp cross-linking, which in turn affects OMV production.

We further examined the dependence of Lpp covalent cross-linking on the Δ*nlpI* hypervesiculation phenotype by quantifying vesiculation of the Δ*ycfS*Δ*ybiS*Δ*erfK*Δ*nlpI* mutant strain. We observed a slight decrease in OMV production of the Δ*ycfS*Δ*ybiS*Δ*erfK*Δ*nlpI* mutant with respect to the Δ*ycfS*Δ*ybiS*Δ*erfK* mutant (Fig. S5B). Since the loss of *nlpI* did not increase OMV production in the triple mutant, these data further support the concept that the Δ*nlpI* hypervesiculation phenotype depends on bound Lpp levels. As of yet, we do not understand the decrease in OMV production of the quadruple mutant with respect to the triple mutant and further work is necessary to elucidate this phenomenon.

## Discussion

Analyses relating envelope architecture and vesiculation phenotypes can help shed light on molecular properties involved in the mechanism of OMV production. Here, we investigated the cause of the strong hypervesiculation phenotype of the Δ*nlpI* mutant strain and found that multiple components of the envelope of this mutant were affected. The data suggest high levels of PG synthesis and decreased levels of covalent Lpp-OM crosslinks for the Δ*nlpI* mutant compared to the WT. The results are consistent with a model in which NlpI is a negative regulator of the PG hydrolases Spr and PBP4, and that the mutant compensates uncontrolled activity of the degradative enzymes with increasing PG synthesis. This increase in PG dynamics could result in the inability to form sufficient Lpp-OM crosslinks. Although deletions in Lpp have been previously shown to alter OMV production (Suzuki et al. 1978; Cascales et al. 2002; Deatherage et al. 2009), the fact that vesiculation can be modulated indirectly via NlpI through changes in PG and consequent detectable differences in bound Lpp levels has not previously been reported.

### Model of NlpI's effect on the bacterial envelope

**Figure 7:**
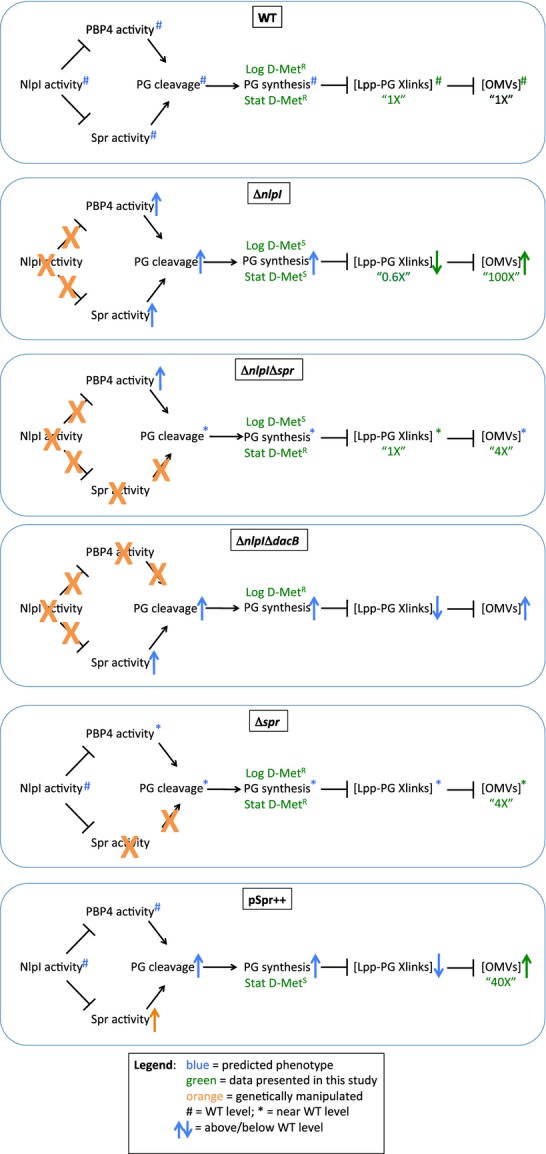
Model of NlpI's regulatory interactions. Proposed model which supports the phenotypic observations. In WT cells, NlpI directly or indirectly controls the endopeptidase activities of PBP4 and Spr to maintain wild-type PG dynamics (cleavage and synthesis), Lpp-PG crosslinks have time to be formed, and basal levels of OMVs are produced. In an *nlpI* mutant, Spr activity is uncontrolled, leading to a “compensatory” increase in PG synthesis, a reduced ability to form Lpp-PG crosslinks, and a consequent increased OMV production. In a *nlpI spr* double mutant, the lack of an increase in Spr activity precludes the “compensatory” increase in PG synthesis in stationary phase, however, uncontrolled PBP4 activity leads to increased PG synthesis in log phase, with relatively minor downstream effects on OMV production. In an *nlpI dacB* double mutant, the lack of PBP4 activity precludes a “compensatory” increase in PG synthesis in log phase, although Spr endopeptidase activity increases, with downstream consequences like those in an *nlpI* mutant. In an *spr* mutant, PG synthesis, Lpp-PG crosslinks, and OMV phenotypes are similar to those of an *nlpI spr* double mutant with the exception of having more controlled PBP4 activity during log phase, although the loss of *spr* may be compensated somewhat by changes in PBP4 activity. When *spr* expression is induced (pSpr++), the effect is similar to that of an *nlpI* mutant, except that PBP4 activity is controlled during log phase. OMV, outer membrane vesicles; WT, wild-type; PG, peptidoglycan.

A model is proposed to explain the phenotypes of the WT and mutant strains described in this study (Fig.7). The pseudo-stationary phase d-Met sensitivity phenotype for the *nlpI* deletion strain is consistent with either NlpI repressing PG endopeptidase activity or by increasing PG synthesis, since either upregulated PG turnover or a reduction in PG synthesis would render cells sensitive to a PG synthesis inhibitor. These interactions could be direct or indirect. The repression model is supported by the data demonstrating that strong overexpression of Spr, but not the mutant Spr lacking endopeptidase activity, leads to similar phenotypes as the Δ*nlpI* mutant. For log-phase cells, NlpI does not act through *spr*, instead it acts through *dacB* to modulate PG dynamics.

It is likely that an increase in PG degradation and synthesis could be accompanied by decreases in Lpp cross-linking levels. There may simply not be enough time to establish a WT concentration of covalently cross-linked Lpp in the mutant cell envelope, since cross-linking is known to be a relatively slow process (Hiemstra et al. 1986). Also a minor activity of Spr has been shown to be the cleavage between mDAP-d-Ala crosslinks between PG stem peptides, resulting in tripeptides (Singh et al. 2012), and these are predicted to be unable to serve as substrates for Lpp-PG cross-linking. A consequence of fewer crosslinks would be loosening of the OM, and hyperproduction of OMVs. For a more detailed examination of the modulation of the processes occurring in the various mutant backgrounds based on our working models, see Figure7.

Although induced extrachromosomal expression of functional Spr was substantially more detrimental to growth in d-Met -treated cultures, interestingly, overexpression of both functional Spr and the inactive mutant mSpr caused a growth defect in the absence of d-Met (Fig.5A and B). It is not surprising that increased levels of a PG degradation enzyme have harmful consequences, since PG is essential for viability (Vollmer and Bertsche 2008), but we were rather surprised that the inactive mutant form showed the same phenotype. Considering this result, we propose that mSpr might bind well to PG as a substrate but then interfere with PG homeostasis because of its inability to cleave and therefore dissociate from the PG. Alternatively, the slight growth defect could be related to envelope stress resulting from the increase in protein content, as evident from a hypervesiculation phenotype (Fig.5C) that is associated with overexpressed envelope proteins (Schwechheimer and Kuehn 2013).

### Insight into OMV production and regulation by WT cells

How might these data on mutant phenotypes and suppressors elucidate the mechanism and modulation of OMV production by WT cells? The data presented here indicate that one route to alter vesiculation is through modulation of PG structure and consequently bound Lpp. We propose that in WT cells the equilibrium between PG synthesis and degradation can determine the concentration of covalently cross-linked Lpp, and that bound Lpp levels inversely affect OMV production. Hence, either a localized or large-scale perturbation of the PG remodeling equilibrium would alter bound Lpp levels, and, consequently, levels of OMV production. In normally growing WT bacteria there are likely patches of PG that are actively being remodeled and therefore have an increased likelihood to be sites of constitutive OMV budding due to the lack of LPP-PG crosslinks. The number and location of these patches may vary depending on growth phase. Acting through PBP4 during log phase, and through Spr during stationary phase, NlpI is proposed to modulate (directly or indirectly) at least part of the PG dynamics. Then, to generate a burst of vesiculation in times of environmental stress or to promote virulence factor delivery, WT cells can alter PG-OM cross-linking via a more dramatic and/or longer acting modulation of PG turnover.

The presence of at least one other distinct pathway that leads to OMV production was revealed from the fact that the loss of *nlpI* and *spr* did not suppress the Δ*degP* hypervesiculation phenotype, and that hypervesiculation of the Δ*nlpI* mutant could not be suppressed by a deletion in *nlpA*, *dsbA*, or *bolA* (Figs.1A, and 2), whereas these suppress hypervesiculation in the Δ*degP* mutant (Schwechheimer and Kuehn 2013). This pathway involves protein buildup in the periplasm and no change in overall Lpp-OM cross-linking levels (Schwechheimer et al. 2014).

In sum, our data bring new insights into the complex interactions that govern the structure of the cell envelope and OMV biogenesis. Further work is required to determine whether these principles are conserved throughout Gram-negative bacteria.

## Supporting Information

Data S1 Experimental procedures.

Figure S1 NlpI complementation. Relative fold OMV production in cultures of the indicated strains with either the pTrc (pT) plasmid or the NlpI expression plasmid (pNlpI) grown in LB overnight at 37°C was determined by quantitating OMVs, normalizing to OD_600_, and dividing by OD_600_-normalized OMV production in a WT culture. **P* ≤ 0.05; *n* = 3. Error bars indicate standard error of the mean (SEM). **Figure S2.** Deletion of *pbpG* does not suppress the phenotypes of the Δ*nlpI* mutant. (A) Relative fold OMV production in cultures of the indicated strains grown in LB overnight at 37°C was determined by quantitating OMVs, normalizing to OD_600_, and dividing by OD_600_-normalized OMV production in a WT culture. **P* ≤ 0.05; *n* = 3. Error bars indicate SEM. (B) Cultures of the indicated strains were grown in LB overnight at 37°C, cells were pelleted and resuspended into fresh LB (*t* = 0) ±10 mmol/L d-Met, and then grown at 37°C. OD_600_ was measured hourly. Data represent the average of two independent experiments. **Figure S3.** Spr-F can induce OMV production. Relative fold OMV production in cultures of the indicated strains grown in LB overnight at 37°C was determined by quantitating OMVs, normalizing to OD_600_, and dividing by OD_600_-normalized OMV production in a WT culture. **P* ≤ 0.05; *n* = 3. Error bars indicate SEM. **Figure S4.** Localization and expression of Spr-F and mSpr-F. (A and B) Anti-FLAG immunoblots of whole cells (lane 1) and cell fractions (lanes 2–7) of Δ*spr* pSpr-FLAG and Δ*spr* pmSpr-FLAG cultures induced with 500 *μ*mol/L IPTG separated using SDS-PAGE. Molecular weight standards (M) are highlighted: *, 25 kDa; >, 20 kDa. Fractions were prepared as described previously (Kesty, N. C., and M. J. Kuehn. 2004. Incorporation of heterologous outer membrane and periplasmic proteins into *Escherichia coli* outer membrane vesicles. *J. Biol. Chem*. 279:2069–2076). **Figure S5.** Δ*nlpI* hypervesiculation depends on the loss of bound Lpp. (A) Anti-Lpp immunoblot of whole cell and copurified PG of the indicted strains. Composite figure created from the rearrangement of lanes from a single immunoblot. (B) Relative fold OMV production in cultures of the indicated strains grown in LB overnight at 37°C was determined by quantitating OMVs, normalizing to OD_600_, and dividing by OD_600_-normalized OMV production in a WT culture. **P* ≤ 0.05; *n* = 3. Error bars indicate SEM.

**Table S1** Summary of growth and membrane integrity phenotypes of mutant and treated cultures. **Table S2** Strains used only in the supplemental material.

## References

[b1] Aramini JM, Rossi P, Huang YJ, Zhao L, Jiang M, Maglaqui M (2008). Solution NMR structure of the NlpC/P60 domain of lipoprotein Spr from *Escherichia coli*: structural evidence for a novel cysteine peptidase catalytic triad. Biochemistry.

[b2] Baba T, Ara T, Hasegawa M, Takai Y, Okumura Y, Baba M (2006). Construction of *Escherichia coli* K-12 in-frame, single-gene knockout mutants: the Keio collection. Mol. Syst. Biol.

[b3] Bardwell JC, Lee JO, Jander G, Martin N, Belin D, Beckwith J (1993). A pathway for disulfide bond formation in vivo. Proc. Natl. Acad. Sci. USA.

[b4] Berleman J, Auer M (2013). The role of bacterial outer membrane vesicles for intra- and interspecies delivery. Environ. Microbiol.

[b5] Beveridge TJ (1999). Structures of gram-negative cell walls and their derived membrane vesicles. J. Bacteriol.

[b6] Braun V, Rehn K (1969). Chemical characterization, spatial distribution and function of a lipoprotein (murein-lipoprotein) of the *E. coli* cell wall. The specific effect of trypsin on the membrane structure. Eur. J. Biochem.

[b7] Braun V, Wolff H (1975). Attachment of lipoprotein to murein (peptidoglycan) of *Escherichia coli* in the presence and absence of penicillin FL 1060. J. Bacteriol.

[b8] Caparros M, Pisabarro AG, de Pedro MA (1992). Effect of D-amino acids on structure and synthesis of peptidoglycan in *Escherichia coli*. J. Bacteriol.

[b9] Cascales E, Bernadac A, Gavioli M, Lazzaroni JC, Lloubes R (2002). Pal lipoprotein of *Escherichia coli* plays a major role in outer membrane integrity. J. Bacteriol.

[b10] Cherepanov PP, Wackernagel W (1995). Gene disruption in *Escherichia coli*: TcR and KmR cassettes with the option of Flp-catalyzed excision of the antibiotic-resistance determinant. Gene.

[b11] Cowles CE, Li Y, Semmelhack MF, Cristea IM, Silhavy TJ (2011). The free and bound forms of Lpp occupy distinct subcellular locations in *Escherichia coli*. Mol. Microbiol.

[b12] Deatherage BL, Cookson BT (2012). Membrane vesicle release in bacteria, eukaryotes, and archaea: a conserved yet underappreciated aspect of microbial life. Infect. Immun.

[b13] Deatherage BL, Lara JC, Bergsbaken T, Rassoulian Barrett SL, Lara S, Cookson BT (2009). Biogenesis of bacterial membrane vesicles. Mol. Microbiol.

[b14] Ellis TN, Kuehn MJ (2010). Virulence and immunomodulatory roles of bacterial outer membrane vesicles. Microbiol. Mol. Biol. Rev.

[b15] Freire P, Vieira HL, Furtado AR, de Pedro MA, Arraiano CM (2006). Effect of the morphogene bolA on the permeability of the *Escherichia coli* outer membrane. FEMS Microbiol. Lett.

[b16] Freire P, Moreira RN, Arraiano CM (2009). BolA inhibits cell elongation and regulates MreB expression levels. J. Mol. Biol.

[b17] Hara H, Abe N, Nakakouji M, Nishimura Y, Horiuchi K (1996). Overproduction of penicillin-binding protein 7 suppresses thermosensitive growth defect at low osmolarity due to an spr mutation of *Escherichia coli*. Microb. Drug Resist.

[b18] Hiemstra H, de Hoop MJ, Inouye M, Witholt B (1986). Induction kinetics and cell surface distribution of *Escherichia coli* lipoprotein under lac promoter control. J. Bacteriol.

[b19] Kamitani S, Akiyama Y, Ito K (1992). Identification and characterization of an *Escherichia coli* gene required for the formation of correctly folded alkaline phosphatase, a periplasmic enzyme. EMBO J.

[b20] Kulp A, Kuehn MJ (2010). Biological functions and biogenesis of secreted bacterial outer membrane vesicles. Annu. Rev. Microbiol.

[b21] Lam H, Oh DC, Cava F, Takacs CN, Clardy J, de Pedro MA (2009). D-amino acids govern stationary phase cell wall remodeling in bacteria. Science.

[b22] MacDonald IA, Kuehn MJ (2013a). Offense and defense: microbial membrane vesicles play both ways. Res. Microbiol.

[b23] MacDonald IA, Kuehn MJ (2013b). Stress-induced outer membrane vesicle production by Pseudomonas aeruginosa. J. Bacteriol.

[b24] Magnet S, Bellais S, Dubost L, Fourgeaud M, Mainardi JL, Petit-Frere S (2007). Identification of the L, D-transpeptidases responsible for attachment of the Braun lipoprotein to *Escherichia coli* peptidoglycan. J. Bacteriol.

[b25] Manning AJ, Kuehn MJ (2011). Contribution of bacterial outer membrane vesicles to innate bacterial defense. BMC Microbiol.

[b26] Maredia R, Devineni N, Lentz P, Dallo SF, Yu J, Guentzel N (2012). Vesiculation from Pseudomonas aeruginosa under SOS. ScientificWorldJournal.

[b27] McBroom AJ, Kuehn MJ (2007). Release of outer membrane vesicles by Gram-negative bacteria is a novel envelope stress response. Mol. Microbiol.

[b28] McBroom AJ, Johnson AP, Vemulapalli S, Kuehn MJ (2006). Outer membrane vesicle production by *Escherichia coli* is independent of membrane instability. J. Bacteriol.

[b29] McMahon KJ, Castelli ME, Vescovi EG, Feldman MF (2012). Biogenesis of outer membrane vesicles in Serratia marcescens is thermoregulated and can be induced by activation of the Rcs phosphorelay system. J. Bacteriol.

[b30] Ohara M, Wu HC, Sankaran K, Rick PD (1999). Identification and characterization of a new lipoprotein, NlpI, in *Escherichia coli* K-12. J. Bacteriol.

[b31] Ortega J, Iwanczyk J, Jomaa A (2009). *Escherichia coli* DegP: a structure-driven functional model. J. Bacteriol.

[b32] Pierce A, Gillette D, Jones PG (2011). *Escherichia coli* cold shock protein CsdA effects an increase in septation and the resultant formation of coccobacilli at low temperature. Arch. Microbiol.

[b33] Raivio TL, Silhavy TJ (2001). Periplasmic stress and ECF sigma factors. Annu. Rev. Microbiol.

[b34] Romeis T, Holtje JV (1994). Penicillin-binding protein 7/8 of *Escherichia coli* is a DD-endopeptidase. Eur. J. Biochem.

[b35] Schooling SR, Beveridge TJ (2006). Membrane vesicles: an overlooked component of the matrices of biofilms. J. Bacteriol.

[b36] Schwechheimer C, Kuehn MJ (2013). Synthetic effect between envelope stress and lack of outer membrane vesicle production in *Escherichia coli*. J. Bacteriol.

[b37] Schwechheimer C, Sullivan CJ, Kuehn MJ (2013). Envelope control of outer membrane vesicle production in Gram-negative bacteria. Biochemistry.

[b38] Schwechheimer C, Kulp A, Kuehn MJ (2014). Modulation of bacterial outer membrane vesicle production by envelope structure and content. BMC Microbiol.

[b39] Silhavy TJ, Berman ML, Enquist LW, Cold Spring Harbor Laboratory (1984). Experiments with gene fusions.

[b40] Silhavy TJ, Kahne D, Walker S (2010). The bacterial cell envelope. Cold Spring Harb. Perspect. Biol.

[b41] Singh SK, SaiSree L, Amrutha RN, Reddy M (2012). Three redundant murein endopeptidases catalyse an essential cleavage step in peptidoglycan synthesis of *Escherichia coli* K12. Mol. Microbiol.

[b42] Spiess C, Beil A, Ehrmann M (1999). A temperature-dependent switch from chaperone to protease in a widely conserved heat shock protein. Cell.

[b43] Suzuki H, Nishimura Y, Yasuda S, Nishimura A, Yamada M, Hirota Y (1978). Murein-lipoprotein of *Escherichia coli*: a protein involved in the stabilization of bacterial cell envelope. Mol. Gen. Genet.

[b44] Tadokoro A, Hayashi H, Kishimoto T, Makino Y, Fujisaki S, Nishimura Y (2004). Interaction of the *Escherichia coli* lipoprotein NlpI with periplasmic Prc (Tsp) protease. J. Biochem. (Tokyo).

[b45] Tashiro Y, Sakai R, Toyofuku M, Sawada I, Nakajima-Kambe T, Uchiyama H (2009). Outer membrane machinery and alginate synthesis regulators control membrane vesicle production in Pseudomonas aeruginosa. J. Bacteriol.

[b47] Uniprot Consortion (2015). UniProt: a hub for protein information. Nucleic Acids Res.

[b48] Weidel W, Pelzer H (1964). Bagshaped macromolecules – a new outlook on bacterial cell walls. Adv. Enzymol. Relat. Areas Mol. Biol.

[b49] Yonezawa H, Osaki T, Kurata S, Fukuda M, Kawakami H, Ochiai K (2009). Outer membrane vesicles of Helicobacter pylori TK1402 are involved in biofilm formation. BMC Microbiol.

[b50] Zhang Z, Feige JN, Chang AB, Anderson IJ, Brodianski VM, Vitreschak AG (2003). A transporter of *Escherichia coli* specific for l- and d-methionine is the prototype for a new family within the ABC superfamily. Arch. Microbiol.

