# Supplementary material for: NlpI-mediated modulation of outer membrane vesicle production through peptidoglycan dynamics in Escherichia coli
Source: Microbiologyopen. 2015 Mar 8;4(3):375–89. doi: 10.1002/mbo3.244 (PMC4475382; doi:10.1002/mbo3.244)
Supplement: Data S1 — Experimental procedures. [file mbo30004-0375-sd1.docx]

**Supplemental Experimental Procedures**

**Outer Membrane Purification**

OM was prepared as described previously (Kesty and Kuehn, 2004).

**Membrane Integrity Assays**

Membrane integrity assay results that have been summarized in Supplemental Table 1 derive from datasets presented in the Supplemental dataset file.

To assess the effect of mutations on cell viability, cultures (5 mL) were inoculated to an OD_600_ ~0.03, cultures were incubated at 37°C for 6 h, and OD_600_ was measured hourly to assess growth for growth curves. OD_600_ at 6 h for control cultures were compared with those of mutant cultures. Better, similar, or worse growth was noted if we found a difference > 0.1 OD_600_ for at least 3 time points (Supplemental Table 1).

The sensitivity of cells to Actinomycin D was also used to assess membrane integrity defects (Leive, 1965; Schwechheimer and Kuehn, 2013). Actinomycin D (Sigma) dissolved in ethanol was added to 25 mL cultures to a final concentration of 5 μg/mL, cultures were incubated at 37° and OD_600_ was measured. Cultures were kept in the dark since Actinomycin D is light sensitive. OD_600_ at 6 h for WT (6h) was compared with those of mutant cultures. Better, similar, or worse growth was noted if we found a difference > 0.1 OD_600_ for at least 3 time points (Supplemental Table 1).

The ability of Sytox Green to enter cells, bind DNA, and consequently fluoresce was used as another assay to assess membrane integrity (Cowles et al., 2011; Schwechheimer and Kuehn, 2013)Untreated 5 mL cultures were grown overnight (37°C, 16-18 h). Cultures (1mL) were centrifuged (Microcentrifuge, 16 000 *g*), resuspended in 600 μL 10 mM Tris-HCl, pH 8.0, and 3 μM Sytox Green (Invitrogen) dissolved in DMSO was added. The mixture was incubated in the dark for 10 min at room temperature and fluorescence was measured (excitation: 500 nm, emission: 550 nm). The fluorescence values were divided by the OD_600_ of the original culture and this value was divided by the fluorescence of the WT control strain to determine relative fold fluorescence change (Supplemental Table 1).

A ToxiLight bioassay kit (Lonza) was used to assess membrane integrity by detecting the amount of adenylate kinase in the culture supernatant (Jacobs et al., 2013; Schwechheimer and Kuehn, 2013). Cultures (5 mL) were grown overnight (37°C, ~16 h). All samples were diluted 10-fold with LB, and 100 μl was placed in a white 96 well plate (in duplicate). To prepare heat killed cells for a positive control, a 5 mL culture was grown overnight (stationary phase) or to an OD_600_ of ~0.4 (log phase), pelleted in a microfuge (10 000 *g*, 5 min, room temperature)*,* resuspended in 1 mL sterile deionized water, and boiled for 3 min followed by sterile filtering (0.45 μm Ultra-free spin column filters, Millipore); lysates were diluted 100-fold and 100 μl was placed in a white 96 well plate (in duplicate). To all sample wells, 100 μl of ToxiLight reagent was added, and the mixture incubated at room temperature for 30 min. Luminescence was measured with a Molecular Devices SpectraMAX GeminiXS spectrometer. The average value of the duplicate sample Luminescence Units (LU) was multiplied by the dilution factor and divided by the OD_600_ value of the original culture to account for differences in culture density (LU/OD). The adenylate kinase concentration was considered below detection (BD) when the measurement was negative (Supplemental Table 1).

**Supplemental Table 1:** Summary of growth and membrane integrity phenotypes of mutant and treated cultures.

| **Strains/ Treatments^1^** | **OMV Production^2^** | **Growth^3^** | **Growth w/ Actinomycin D^4^** | **Sytox Green^5^** | **Adenylate Kinase^6^** |
| --- | --- | --- | --- | --- | --- |
|  |  |  |  |  |  |
| WT pTrc | 1 | WT | WT | WT | ++ |
| Δ*nlpI* pTrc | 150 | WT | WT | >WT | ++ |
| Δ*spr* pTrc | 4 | WT | WT | >WT | ++ |
| Δ*spr* pSpr | 2 | WT | WT | <WT | + |
| Δ*spr* pSpr / 10 μM IPTG | 4 | WT | WT | >WT | ++ |
| Δ*spr* pSpr / 500 μM IPTG | 30 | <WT | <WT | >WT | ++ |
| Δ*spr* pmSpr / 500 μM IPTG | 6 | <WT | <WT | >WT | ++ |
| Δ*spr* pSpr-FLAG | 3 | WT | WT | WT | ++ |
| Δspr pSpr-FLAG / 500 μM IPTG | 52 | <WT | <WT | >WT | ++ |
|  |  |  |  |  |  |
| WT | 1 | WT | WT | WT | ++ |
| Δ*nlpI* | 151 | WT | WT | >WT | ++ |
| Δ*nlpA* | 0.6 | WT | WT | >WT | ++ |
| Δ*nlpA*Δ*nlpI* | 130 | WT | WT | >WT | ++ |
| Δ*bolA* | 0.4 | WT | WT | WT | ++ |
| Δ*nlpI*Δ*bolA* | 287 | WT | WT | >WT | ++ |
| Δ*dsbA* | 0.6 | WT | WT | WT | ++ |
| Δ*nlpI*Δ*dsbA* | 224 | WT | WT | >WT | ++ |
| Δ*spr* | 3.8 | <WT | WT | WT | ++ |
| Δ*nlpI*Δ*spr* | 4 | <WT | WT | >WT | ++ |
| Δ*nlpI*Δ*nlpC* | 333 | WT | WT | >WT | + |
| Δ*nlpI*Δ*ydhO* | 345 | WT | WT | >WT | ++ |
| Δ*nlpI*Δ*yafL* | 369 | WT | WT | >WT | ++ |
| Δ*nlpI*Δ*yebA* | 348 | WT | WT | >WT | ++ |
| Δ*pbpG* | 1 | WT | WT | WT | BD |
| Δ*nlpI*Δ*pbpG* | 100 | WT | WT | >WT | BD |
| Δ*degP* | 56 | <WT | WT | WT | ++ |
| Δ*spr*Δ*degP* | 72 | <WT | WT | WT | ++ |
| Δ*nlpI*Δ*spr*Δ*degP* | 106 | <WT | WT | >WT | ++ |

^1^Sets of assays performed in the same experimental group are separated by a space in the table; heat-killed cells were used as a positive control for Sytox Green and adenylate kinase activity; sup, spent culture supernatant; each assay was repeated at least twice.

^2^Fold-change in OMV production relative to WT or WT control. See Figures for statistical evaluations.

^3^Growth in LB: WT, like WT or WT control; <WT, below WT or WT control.

^4^Growth in LB with 5 μg/mL Actinomycin D: WT, like WT or WT control; <WT, below WT or WT control.

**^5^**Sytox Green entry: WT, no significant difference from WT or WT control; <WT, significantly less than WT or WT control; >WT, significantly higher compared to WT or WT control.

**^6^**Adenylate kinase in cell-free supernatant:+, ~100-999 LU/OD; ++, ~1000-9999 LU/OD; For comparison, heat killed cells yielded >40,000 LU/OD for log phase cells and >20,000 LU/OD for stationary phase; BD, below detection, refers to samples with a negative value.

**Supplemental Table 2: Strains used only in the supplemental material**

**Supplemental References:**

Cowles, C.E., Li, Y., Semmelhack, M.F., Cristea, I.M., and Silhavy, T.J. (2011). The free and bound forms of Lpp occupy distinct subcellular locations in Escherichia coli. Mol Microbiol *79*, 1168-1181.

Jacobs, A.C., Didone, L., Jobson, J., Sofia, M.K., Krysan, D., and Dunman, P.M. (2013). Adenylate kinase release as a high-throughput-screening-compatible reporter of bacterial lysis for identification of antibacterial agents. Antimicrob Agents Chemother *57*, 26-36.

Kesty, N.C., and Kuehn, M.J. (2004). Incorporation of heterologous outer membrane and periplasmic proteins into Escherichia coli outer membrane vesicles. J Biol Chem *279*, 2069-2076.

Leive, L. (1965). Actinomycin Sensitivity in Escherichia Coli Produced by EDTA. Biochem Biophys Res Commun *18*, 13-17.

Schwechheimer, C., and Kuehn, M.J. (2013). Synthetic Effect between Envelope Stress and Lack of Outer Membrane Vesicle Production in Escherichia coli. J Bacteriol *195*, 4161-4173.
