# Supplementary material for: NlpI-mediated modulation of outer membrane vesicle production through peptidoglycan dynamics in Escherichia coli
Source: Microbiologyopen. 2015 Mar 8;4(3):375–89. doi: 10.1002/mbo3.244 (PMC4475382; doi:10.1002/mbo3.244)
Supplement: Figure S1 — NlpI complementation. Relative fold OMV production in cultures of the indicated strains with either the pTrc (pT) plasmid or the NlpI expression plasmid (pNlpI) grown in LB overnight at 37°C was determined by quantitating OMVs, normalizing to OD600, and dividing by OD600-normalized OMV production in a WT culture. *P ≤ 0.05; n = 3. Error bars indicate standard error of the mean (SEM). Figure S2. Deletion of pbpG does not suppress the phenotypes of the ΔnlpI mutant. (A) Relative fold OMV production in cultures of the indicated strains grown in LB overnight at 37°C was determined by quantitating OMVs, normalizing to OD600, and dividing by OD600-normalized OMV production in a WT culture. *P ≤ 0.05; n = 3. Error bars indicate SEM. (B) Cultures of the indicated strains were grown in LB overnight at 37°C, cells were pelleted and resuspended into fresh LB (t = 0) ±10 mmol/L d-Met, and then grown at 37°C. OD600 was measured hourly. Data represent the average of two independent experiments. Figure S3. Spr-F can induce OMV production. Relative fold OMV production in cultures of the indicated strains grown in LB overnight at 37°C was determined by quantitating OMVs, normalizing to OD600, and dividing by OD600-normalized OMV production in a WT culture. *P ≤ 0.05; n = 3. Error bars indicate SEM. Figure S4. Localization and expression of Spr-F and mSpr-F. (A and B) Anti-FLAG immunoblots of whole cells (lane 1) and cell fractions (lanes 2–7) of Δspr pSpr-FLAG and Δspr pmSpr-FLAG cultures induced with 500 μmol/L IPTG separated using SDS-PAGE. Molecular weight standards (M) are highlighted: *, 25 kDa; >, 20 kDa. Fractions were prepared as described previously (Kesty, N. C., and M. J. Kuehn. 2004. Incorporation of heterologous outer membrane and periplasmic proteins into Escherichia coli outer membrane vesicles. J. Biol. Chem. 279:2069–2076). Figure S5. ΔnlpI hypervesiculation depends on the loss of bound Lpp. (A) Anti-Lpp immunoblot of whole cell and copurified PG of the indicted str [file mbo30004-0375-sd2.pdf]

Supporting Information

Sup Fig 1

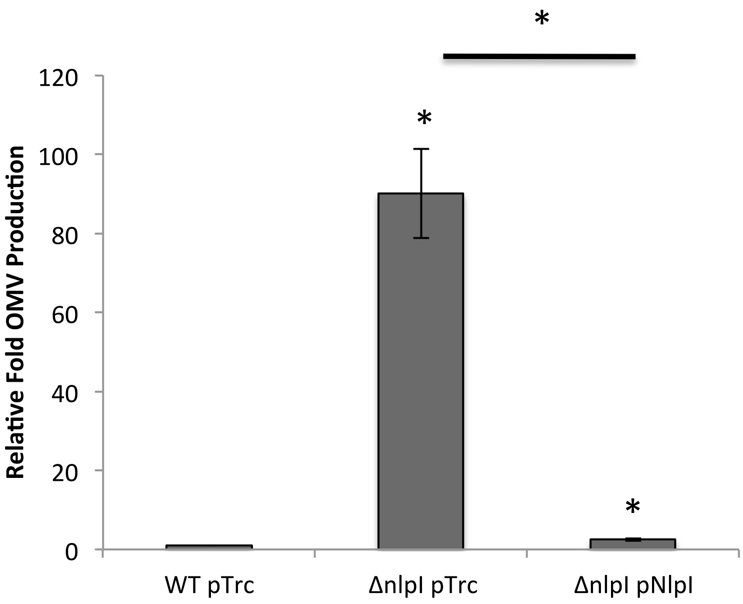

Sup Fig 1 NlpI complementation Relative fold OMV production in cultures of the indicated strains with either the pTrc (pT) plasmid or the NlpI expression plasmid (pNlpI) grown in LB overnight at 37°C was determined by quantitating OMVs, normalizing to OD_{600}, and dividing by OD_{600}-normalized OMV production in a WT culture. *, $p \leq 0.05$; n=3. Error bars indicate standard error of the mean (SEM).

Sup Fig 2

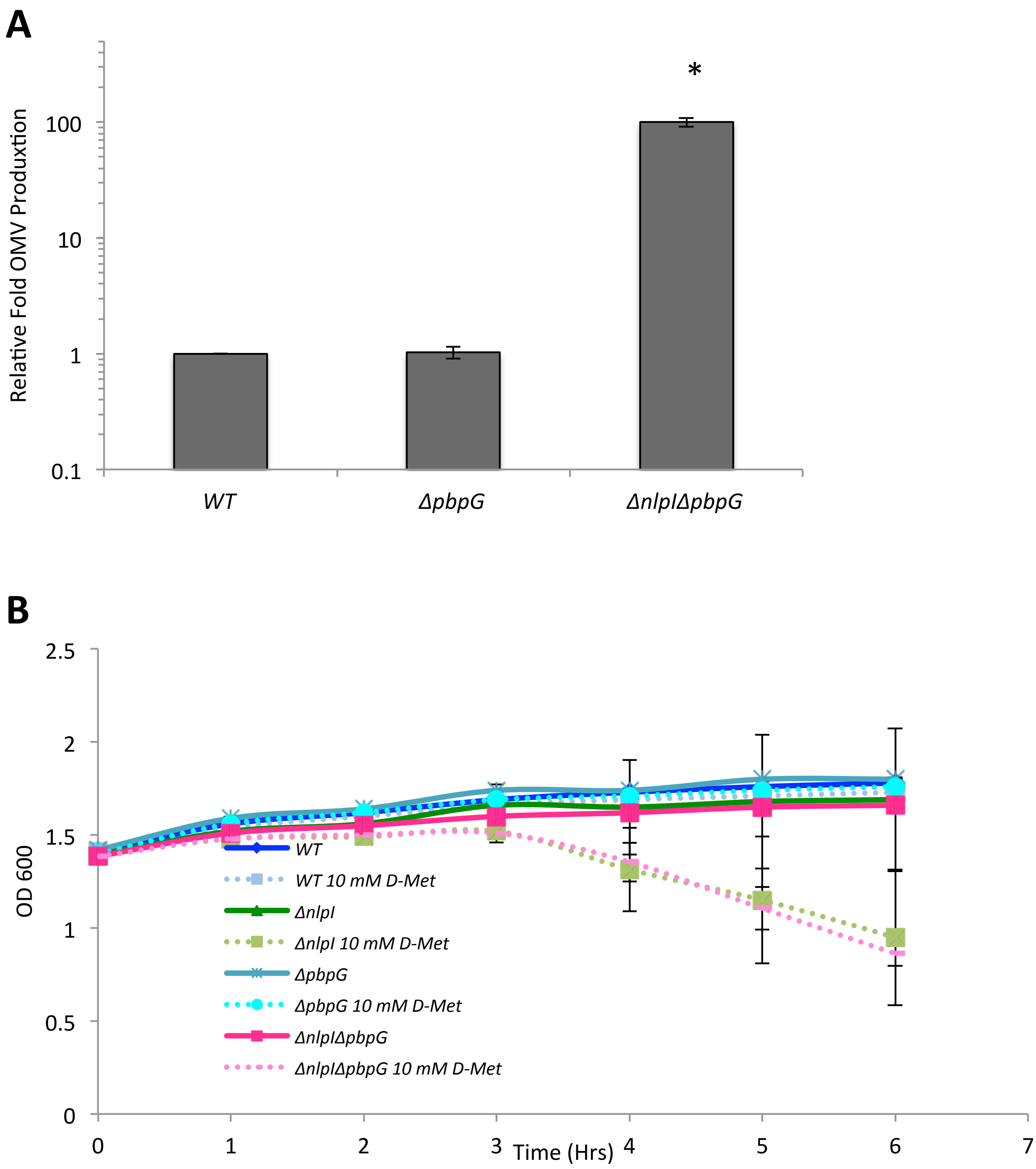

Sup Fig 2 Deletion of *pbpG* does not suppress the phenotypes of the Δ*nlpI* mutant. (A) Relative fold OMV production in cultures of the indicated strains grown in LB overnight at 37°C was determined by quantitating OMVs, normalizing to OD_{600}, and dividing by OD_{600}-normalized OMV production in a WT culture. *, $p \leq 0.05$; n=3. Error bars indicate SEM. (B) Cultures of the indicated strains were grown in LB overnight at 37°C, cells were pelleted and resuspended into fresh LB (t=0) +/- 10 mM D-Met, and then grown at 37°C. OD_{600} was measured hourly. Data represents the average of two independent experiments.

Sup Fig 3

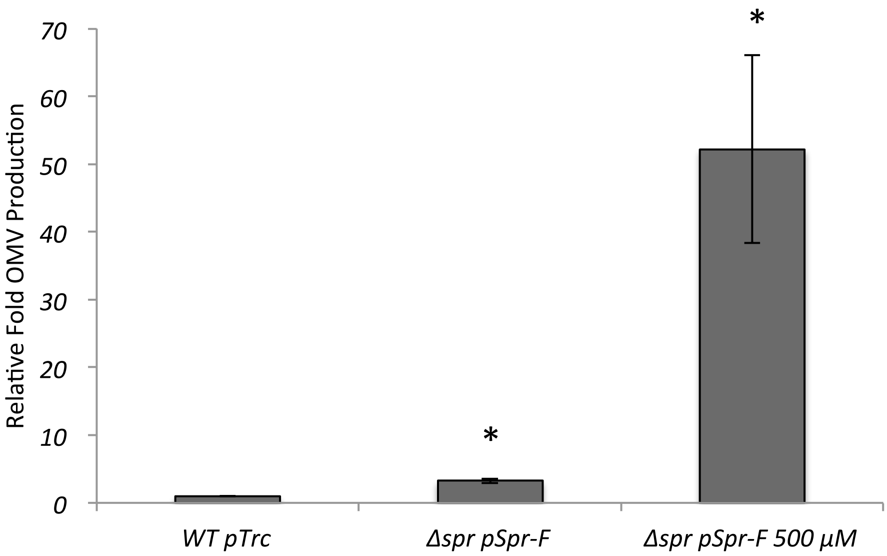

Sup Fig 3 Spr-F can induce OMV Production Relative fold OMV production in cultures of the indicated strains grown in LB overnight at 37°C was determined by quantitating OMVs, normalizing to OD_{600}, and dividing by OD_{600}-normalized OMV production in a WT culture. *, $p \leq 0.05$; n=3. Error bars indicate SEM.

Sup Fig 4

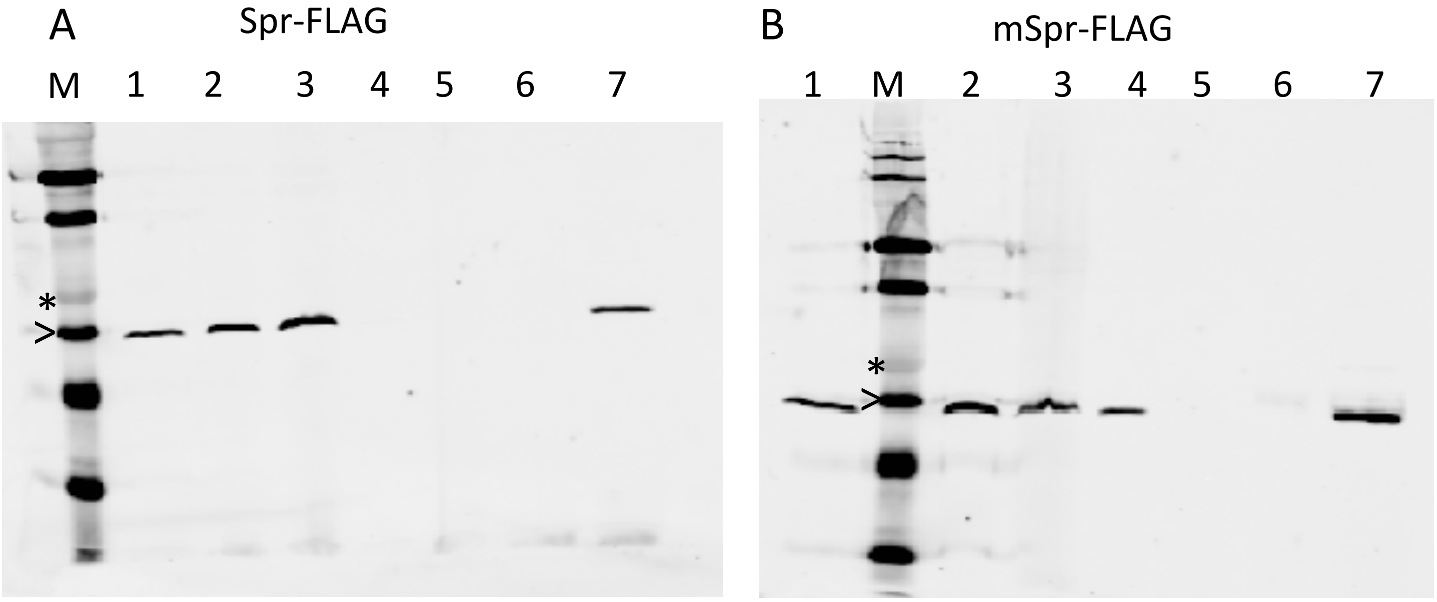

1: Whole Cell
2: Lysate
3: Soluble Fraction
4: Insoluble Fraction
5: Cytoplasm
6: Inner Membrane
7: Outer Membrane

Sup Fig 4 Localization and expression of Spr-F and mSpr-F
(A,B) Anti-FLAG immunoblots of whole cells (lane 1) and cell fractions (lanes 2-7) of Δ*spr* pSpr-FLAG and Δ*spr* pmSpr-FLAG cultures induced with 500 μM IPTG separated using SDS-PAGE. Molecular weight standards (M) are highlighed: *, 25kDa; >, 20 kDa. Fractions were prepared as described previously (Kesty, N.C., and Kuehn, M.J. (2004). Incorporation of heterologous outer membrane and periplasmic proteins into Escherichia coli outer membrane vesicles. J Biol Chem *279*, 2069-2076.)

Sup Fig 5

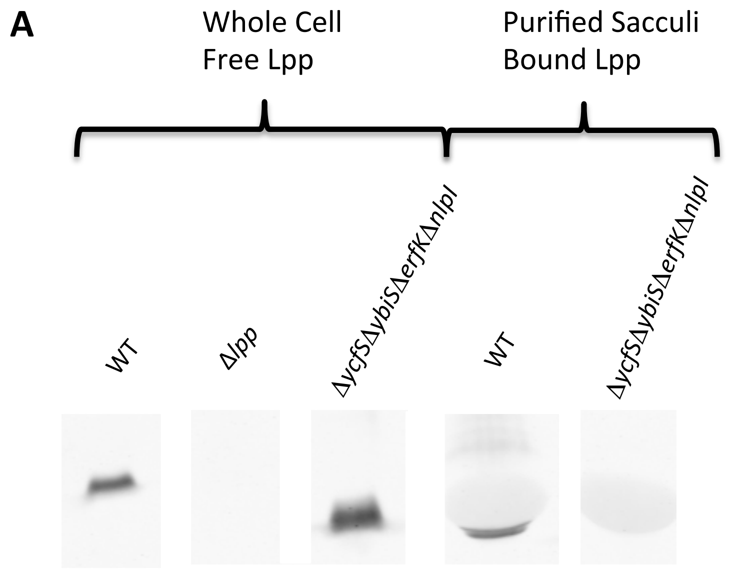

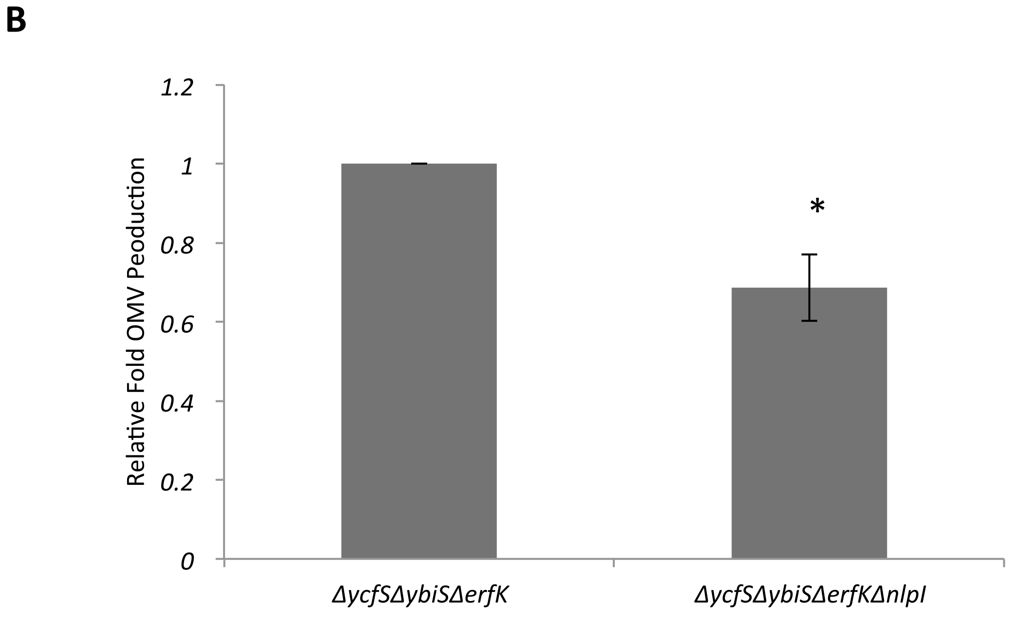

Sup Fig 5 Δ*nlpI* hypervesiculation depends on the loss of bound Lpp (A) Anti-Lpp immunoblot of whole cell and copurified PG of the indicted strains. Composite figure created from the rearrangement of lanes from a single immunoblot. (B) Relative fold OMV production in cultures of the indicated strains grown in LB overnight at 37°C was determined by quantitating OMVs, normalizing to OD_{600}, and dividing by OD_{600}-normalized OMV production in a WT culture. *, $p \leq 0.05$; n=3. Error bars SEM.
