# Supplementary material for: NlpI-mediated modulation of outer membrane vesicle production through peptidoglycan dynamics in Escherichia coli
Source: Microbiologyopen. 2015 Mar 8;4(3):375–89. doi: 10.1002/mbo3.244 (PMC4475382; doi:10.1002/mbo3.244)
Supplement: Supplementary file 3 — Table S1 Summary of growth and membrane integrity phenotypes of mutant and treated cultures. Table S2 Strains used only in the supplemental material. [file mbo30004-0375-sd3.pdf]

Supporting Dataset for membrane integrity data summarized in Sup Table 1

Schwechheimer, Rodriguez and Kuehn

MicrobiologyOpen

Δ*nlpI* pTrc, 37°C

Growth, n=3

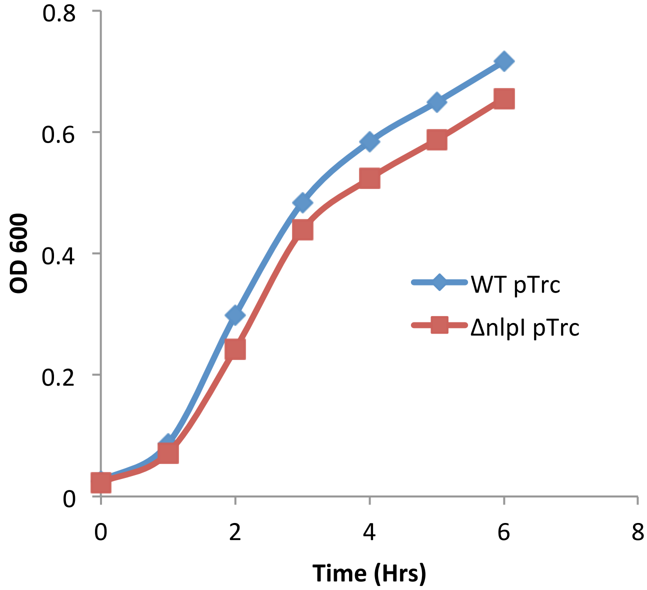

Sytox Green, n=2

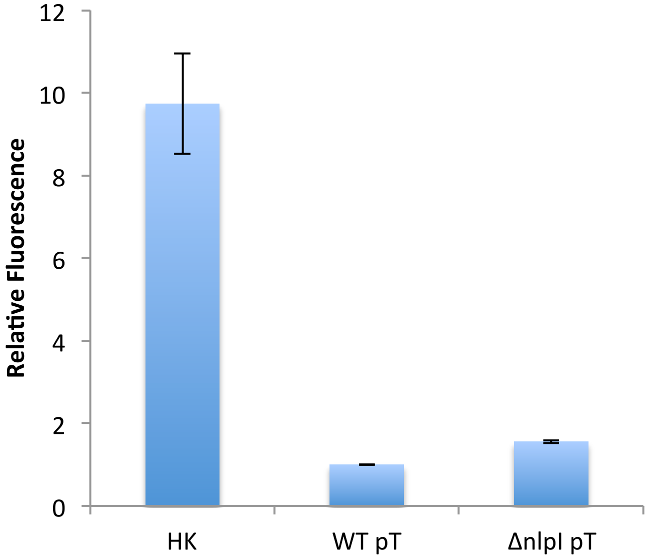

5 µg/ ml Actinomycin D

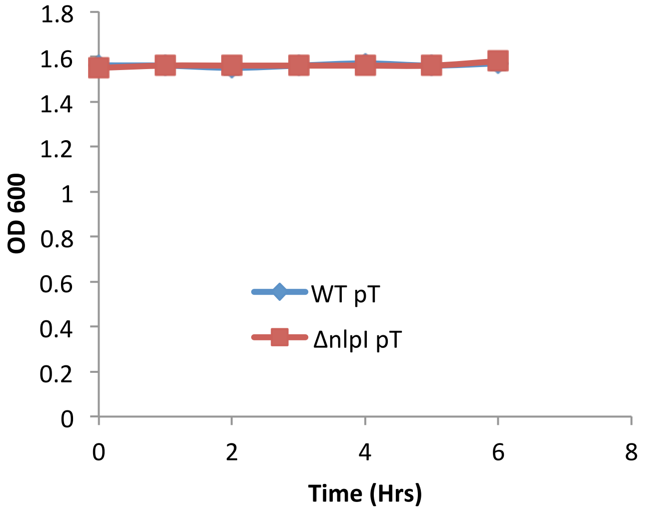

n=2, t=0: Act

Adenylate Kinase

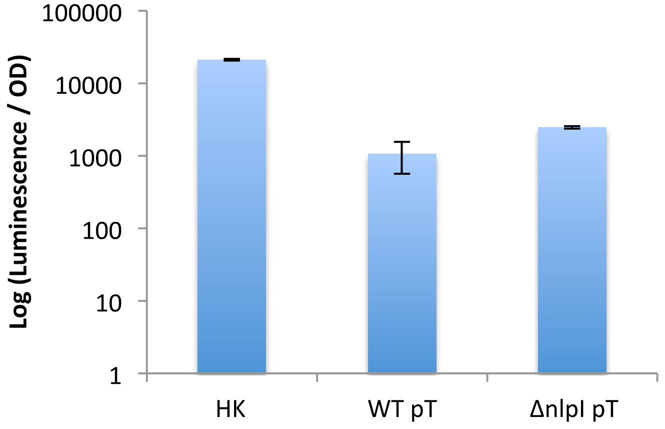

HK, WT, Δ*nlpI* pTrc: n=2

(HK: Heat Killed)

Δ*spr* pTrc, 37°C

Growth, n=3

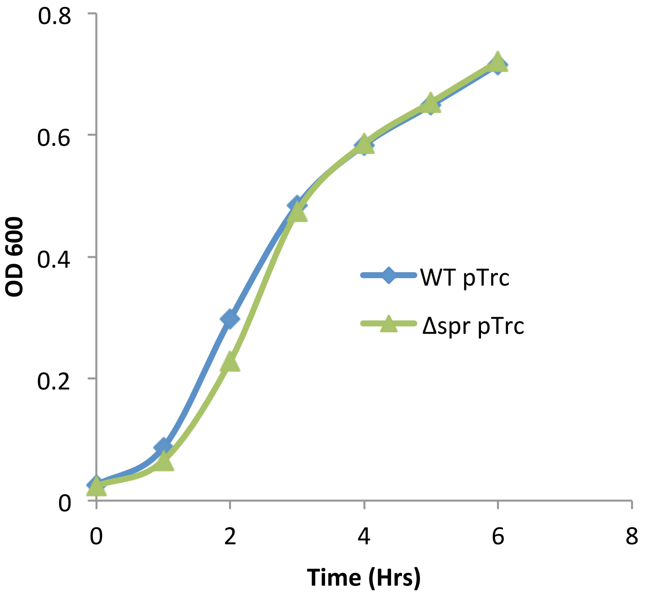

Sytox Green, n=2

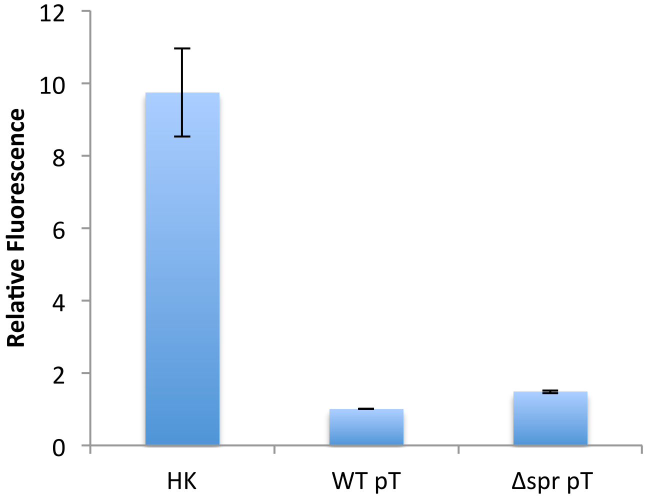

5 µg/ ml Actinomycin D

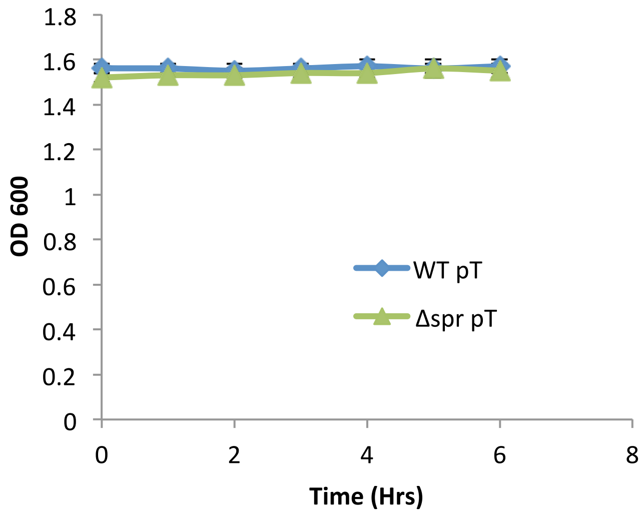

n=2, t=0: Act

Adenylate Kinase

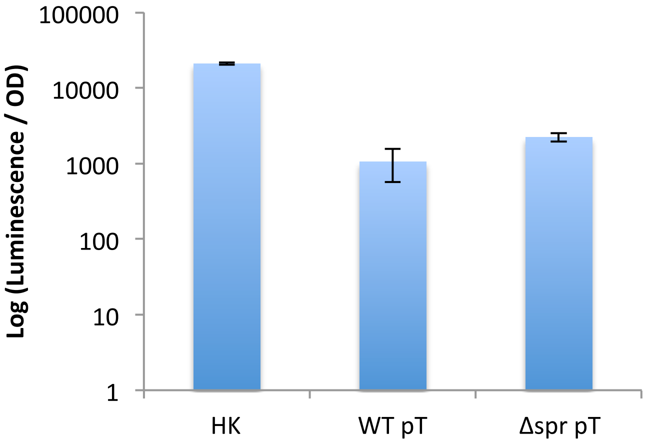

HK, WT, Δ*spr* pTrc: n=2

Δ*spr* Spr, 37°C

Growth, n=3

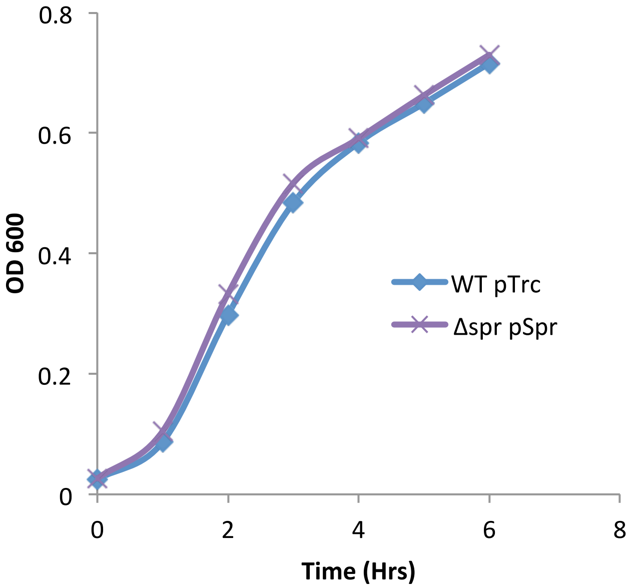

Sytox Green, n=2

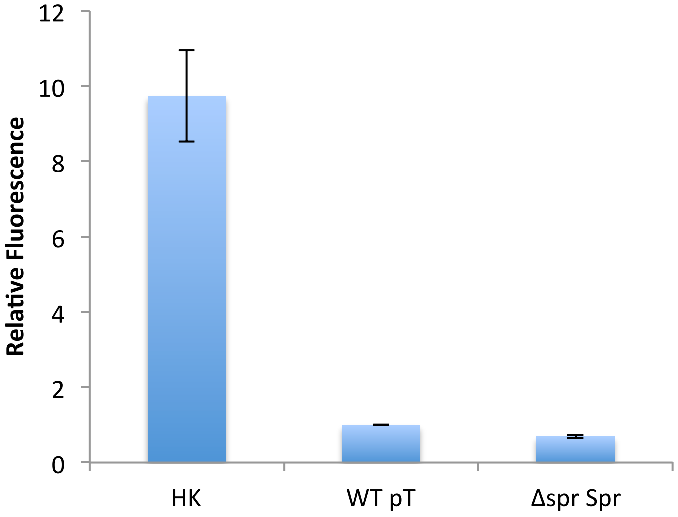

5 µg/ ml Actinomycin D

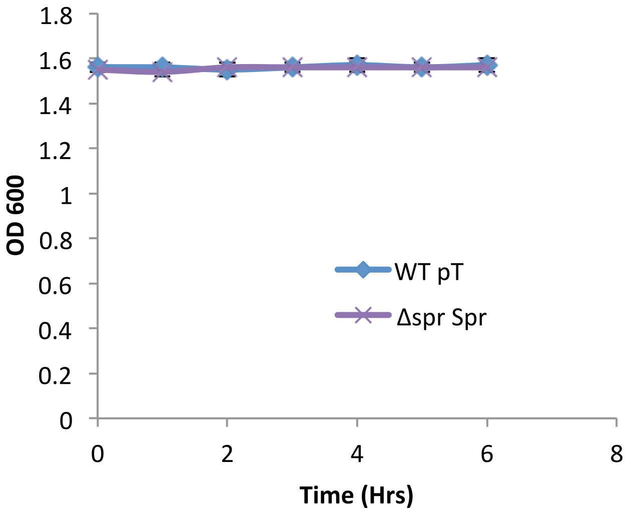

Adenylate Kinase

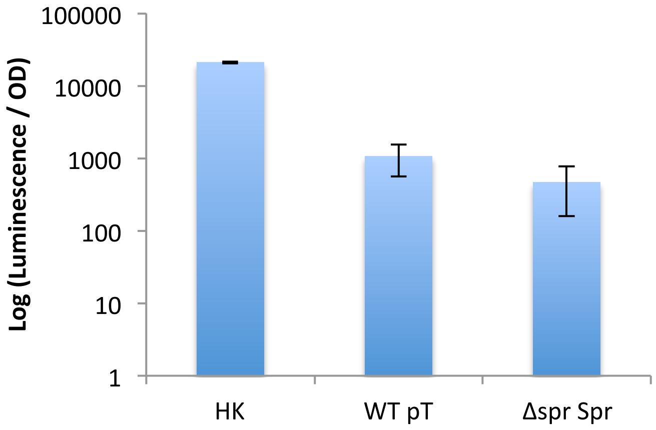

HK, WT, Δ*spr* Spr: n=2

n=2, t=0: Act

Δ*spr* Spr 10 µM, 37°C

Growth, n=3 (induced at inoculation)

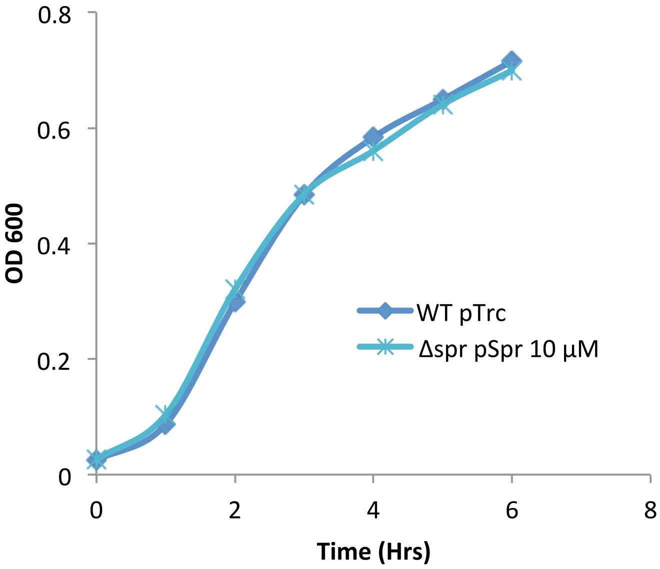

Sytox Green

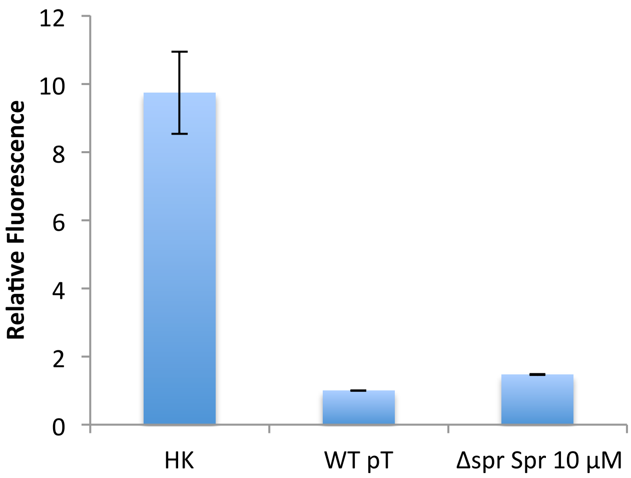

n=2 (induced at inoculation)

5 µg/ ml Actinomycin D

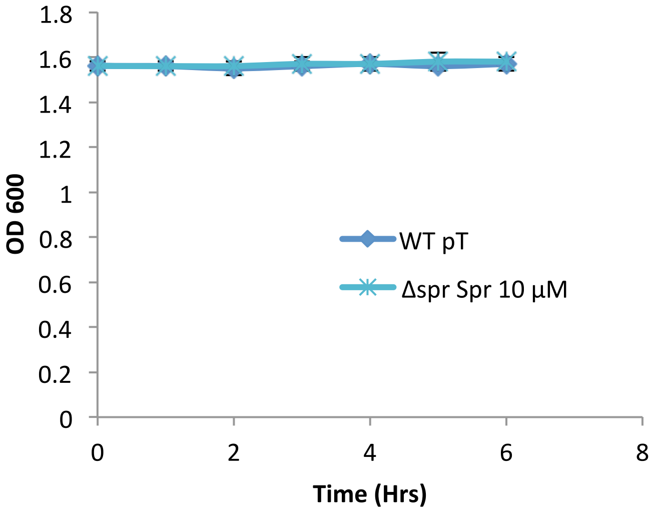

n=2, t=0: Act
(induced at inoculation)

Adenylate Kinase

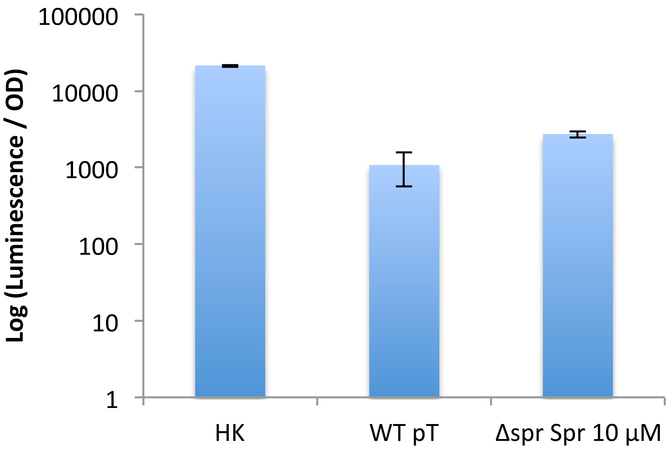

HK, WT, Δ*spr* Spr, 10 µM: n=2
(induced at inoculation)

Δspr Spr 500 μM, 37°C

Growth, n=3 (induced at inoculation)

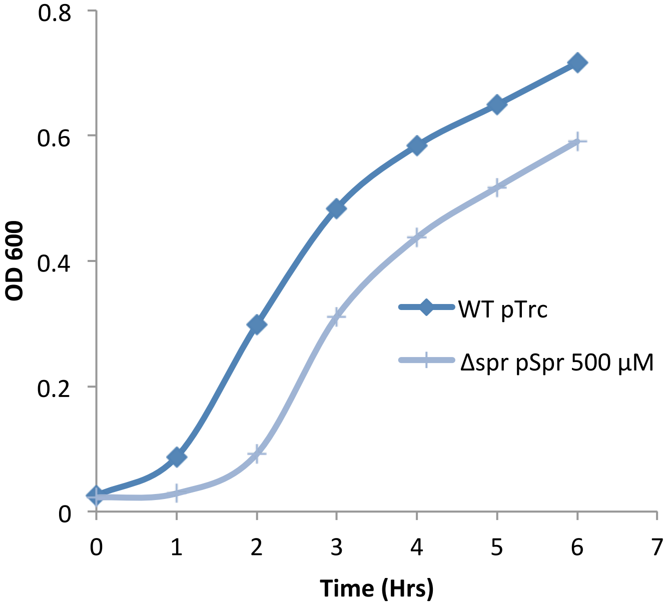

Sytox Green

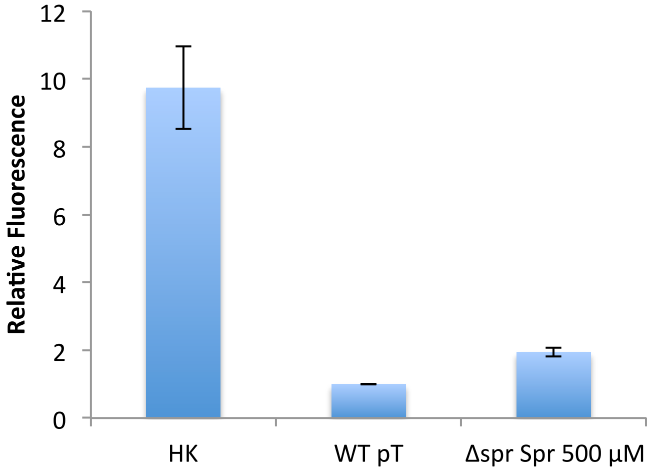

n=2 (induced at inoculation)

5 μg/ ml Actinomycin D

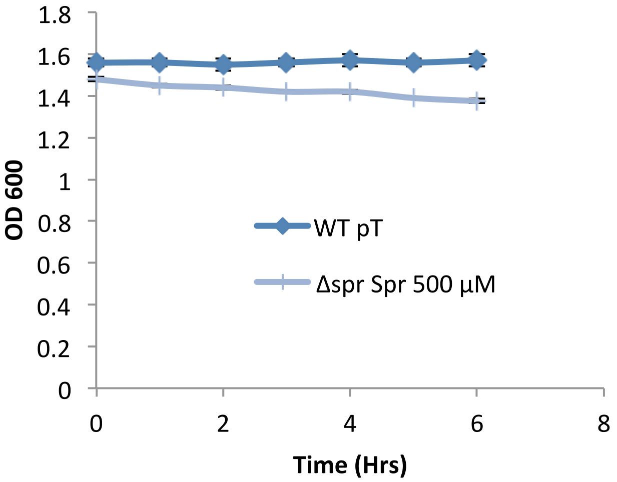

Adenylate Kinase

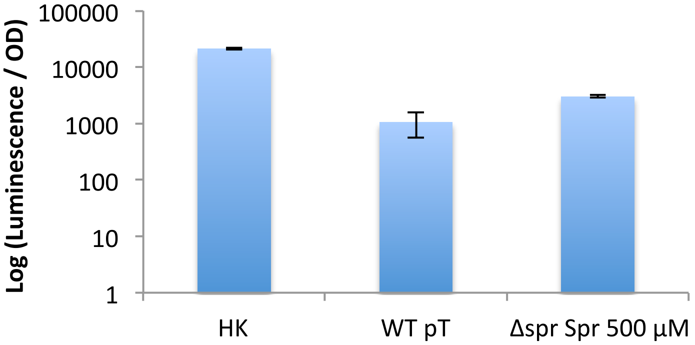

n=2, t=0: Act
(induced at inoculation)

HK, WT, *Δspr* Spr, 500 μM: n=2
(induced at inoculation)

Δ*spr* mSpr 500 µM, 37°C

Growth, n=3 (induced at inoculation)

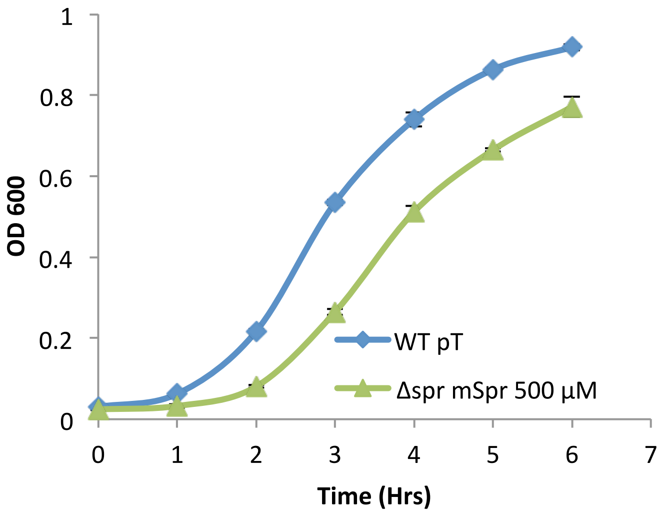

5 µg/ ml Actinomycin D

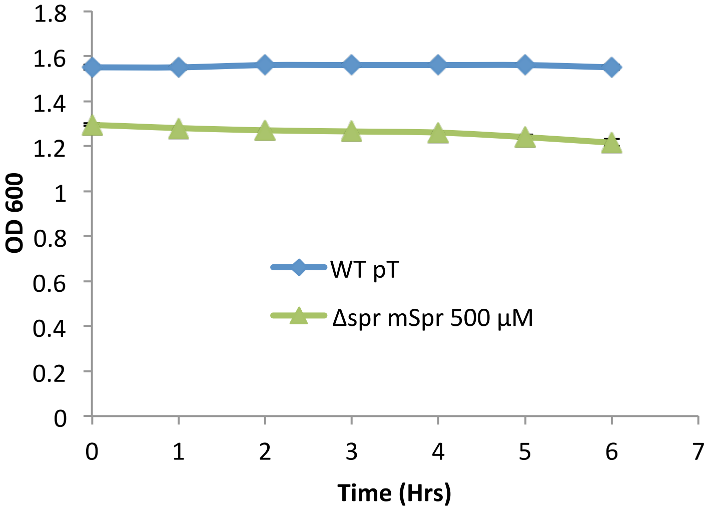

n=2, t=0: Act
(induced at inoculation)

Sytox Green

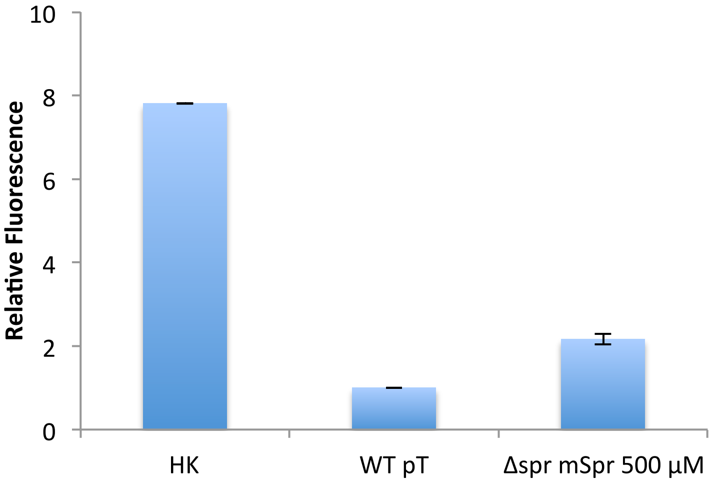

n=2 (induced at inoculation)

Adenylate Kinase

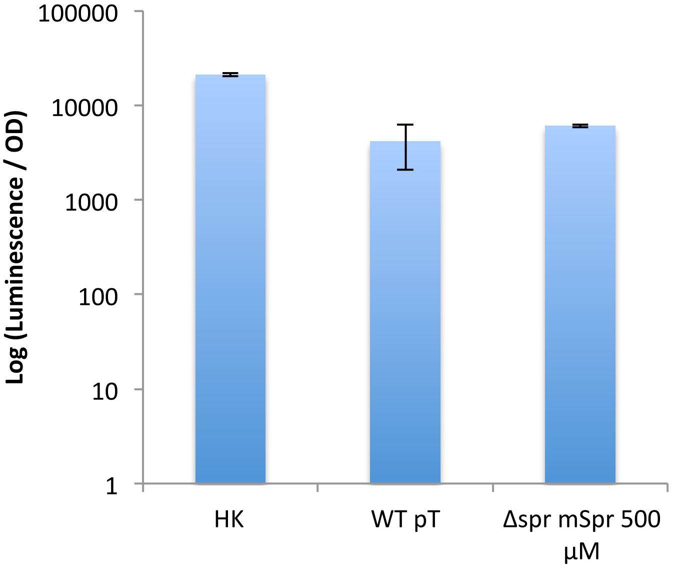

n=2 (induced at inoculation)

Δ*nlpI*, 37°C

Growth, n=3

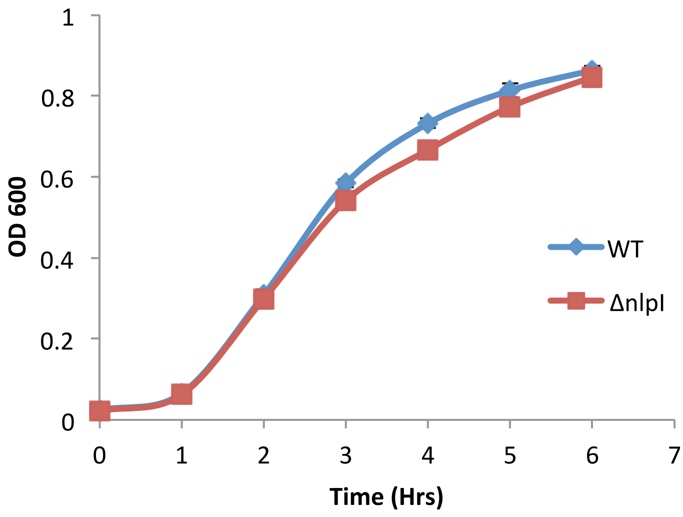

Sytox Green

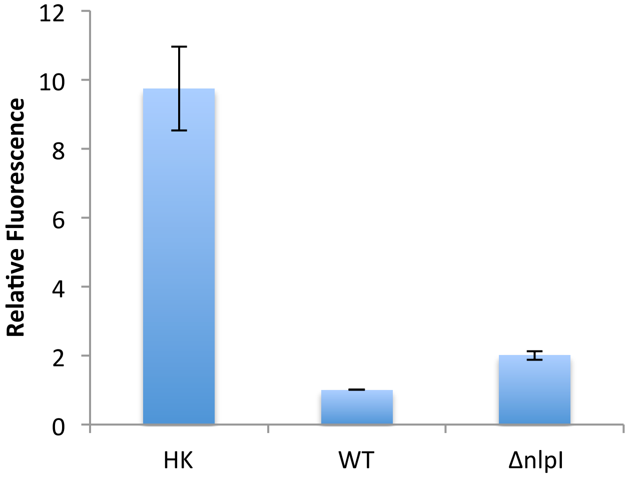

n=2

5 µg/ ml Actinomycin D

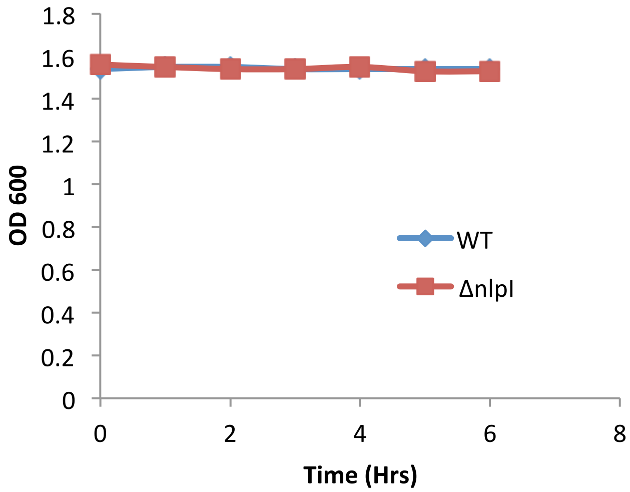

Adenylate Kinase

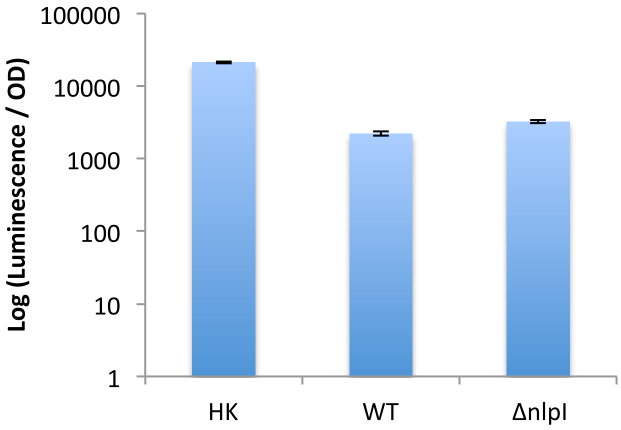

n=2

n=2, t=0: Act

ΔnlpA, 37°C

Growth, n=3

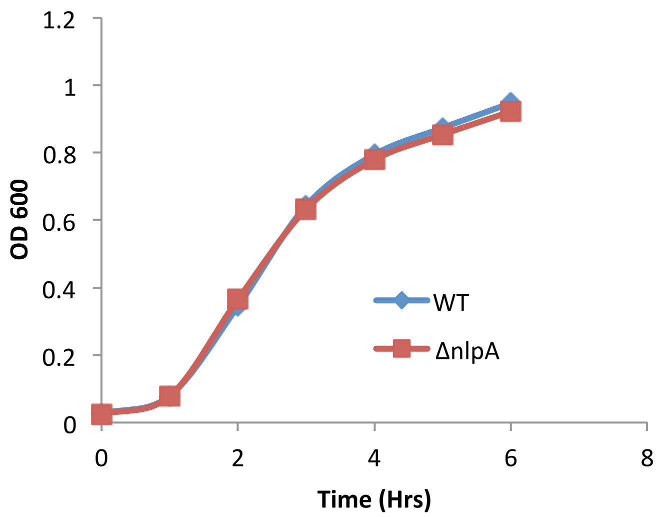

Sytox Green

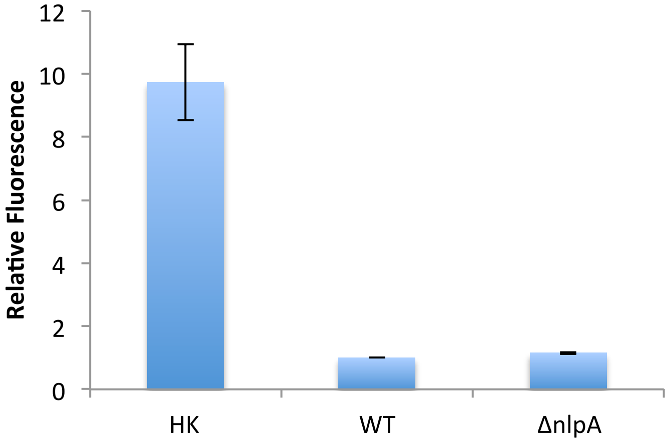

n=2

5 µg/ ml Actinomycin D

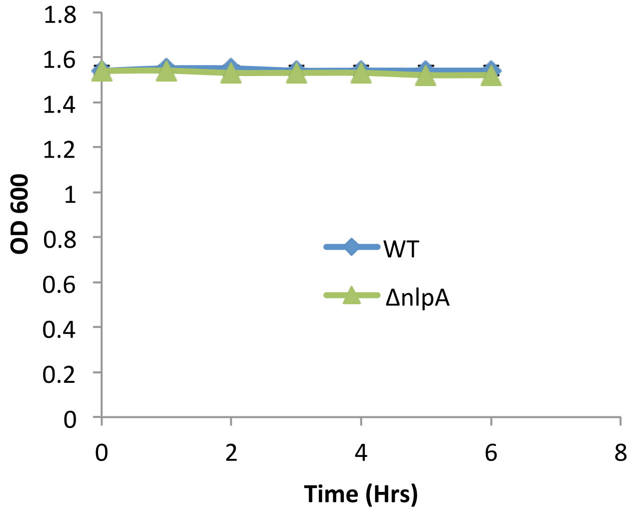

Adenylate Kinase

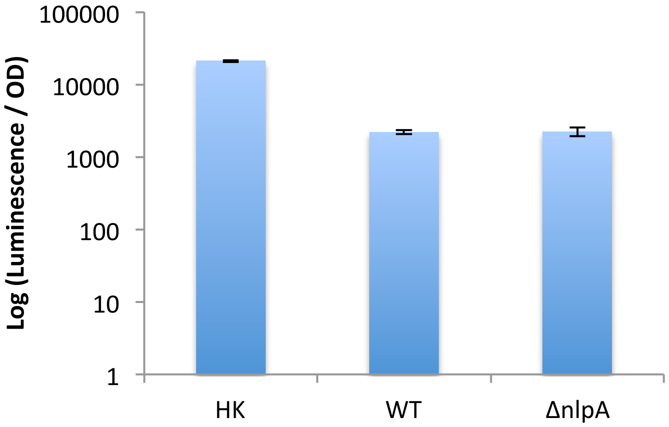

n=2

n=2, t=0: Act

ΔnlpAΔnlpI, 37°C

Growth, n=3

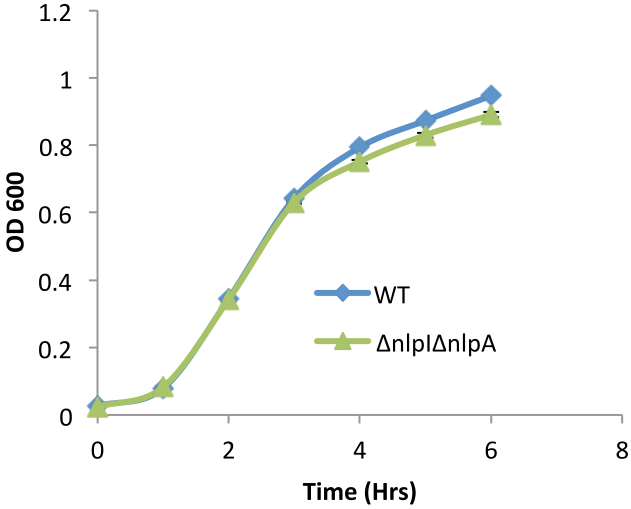

Sytox Green

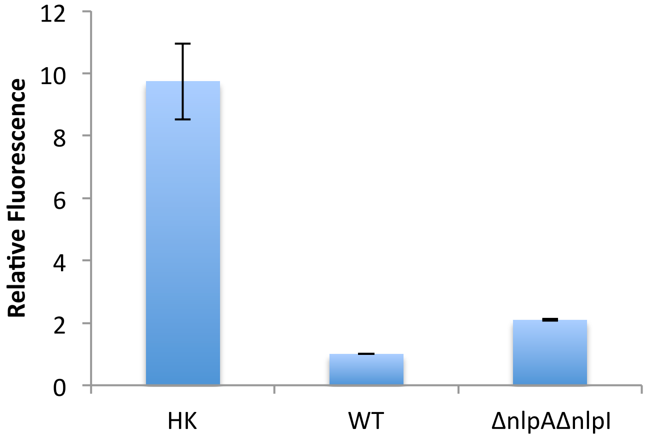

n=2

5 µg/ ml Actinomycin D

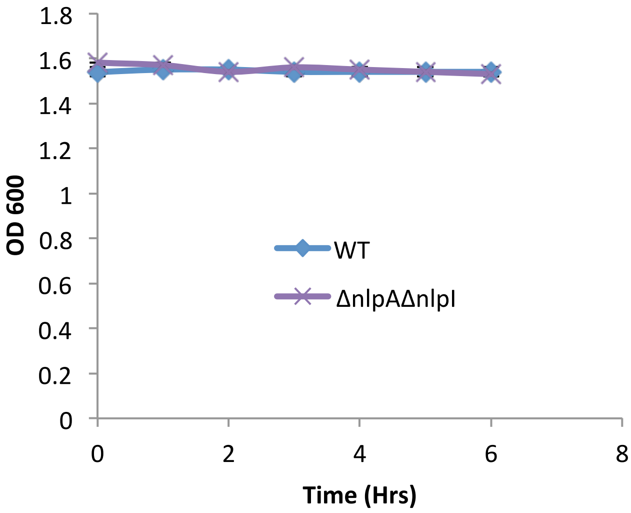

Adenylate Kinase

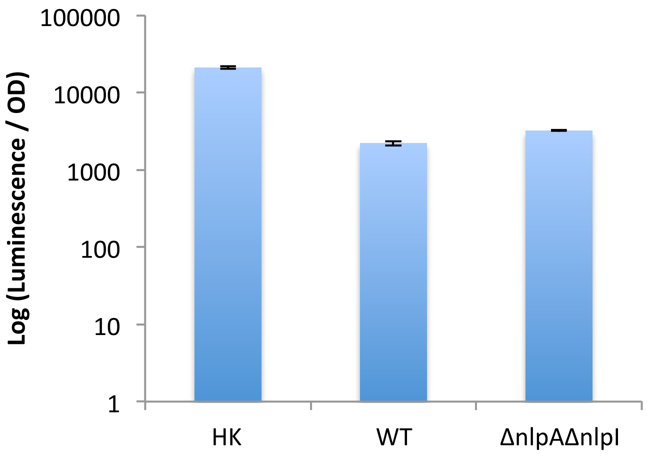

n=2

n=2, t=0: Act

Δ*bolA*, 37°C

Growth, n=3

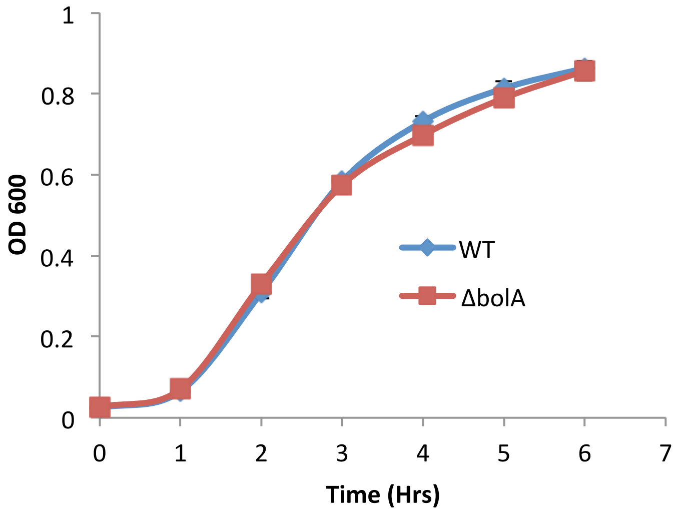

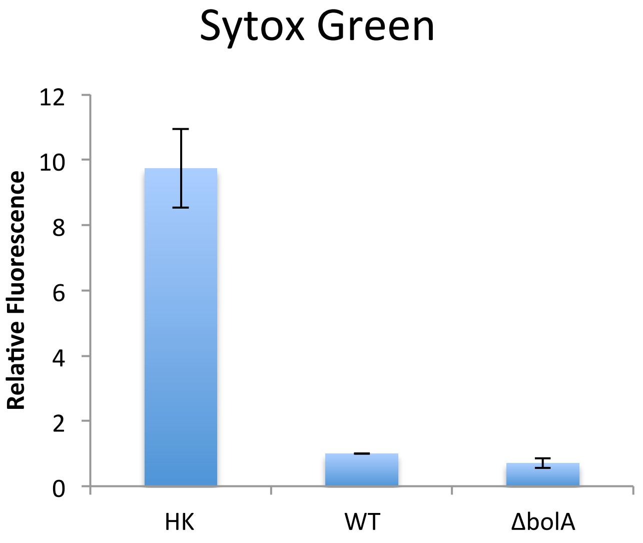

n=2

5 µg/ ml Actinomycin D

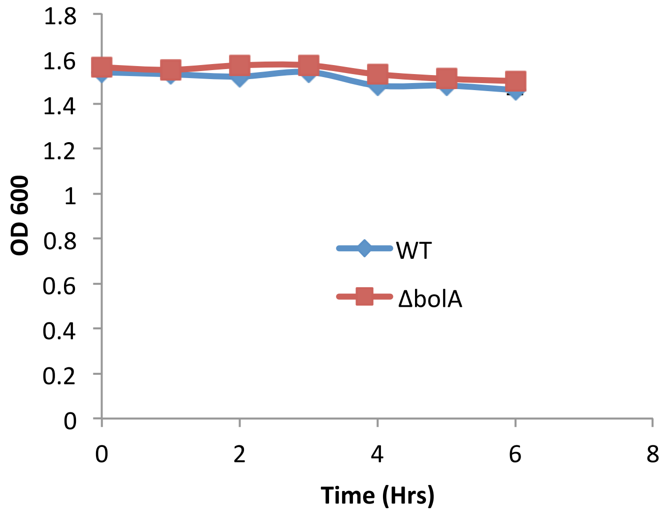

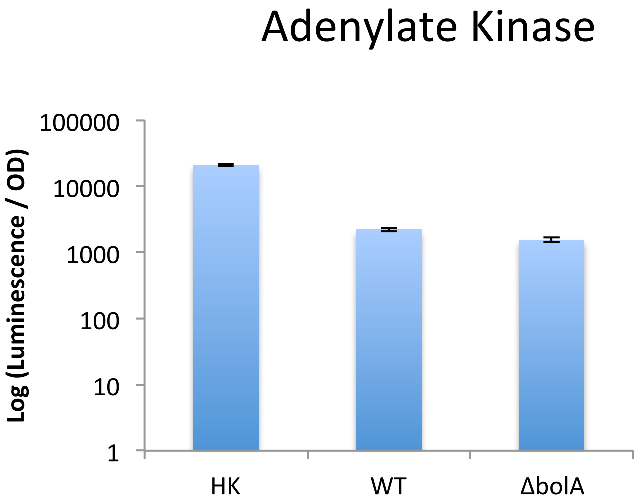

n=2

n=2, t=0: Act

ΔnlpIΔbolA, 37°C

Growth, n=3

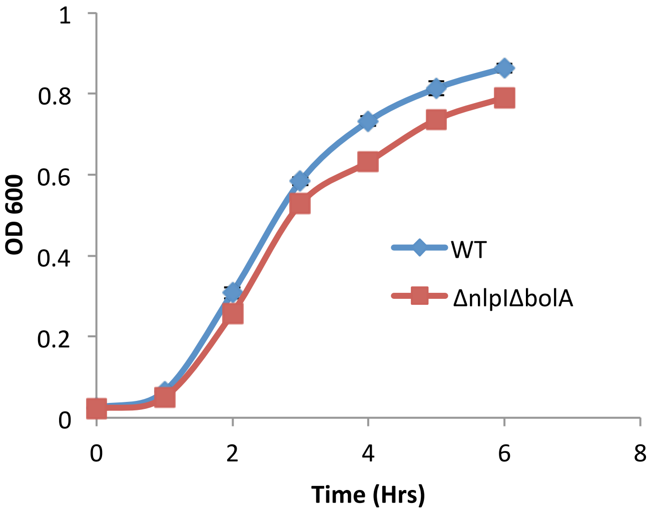

Sytox Green

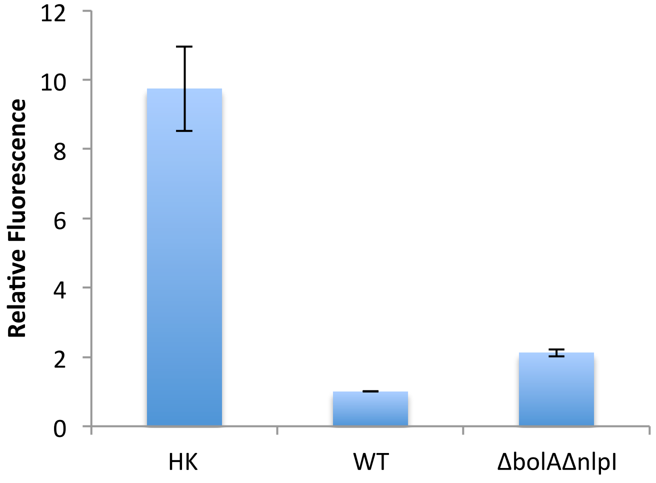

n=2

5 µg/ ml Actinomycin D

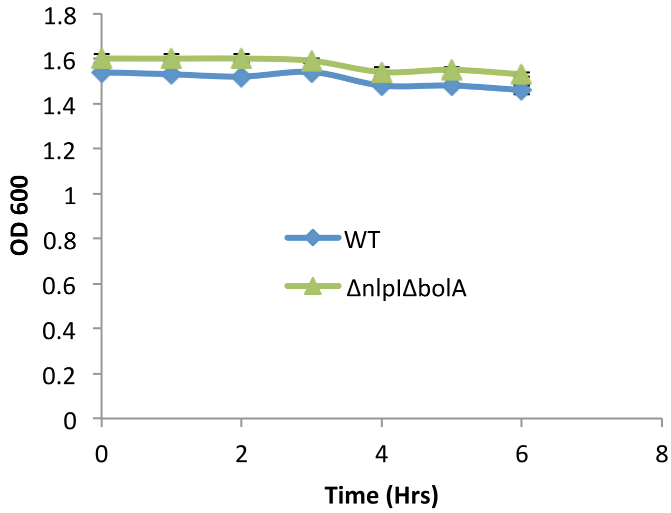

Adenylate Kinase

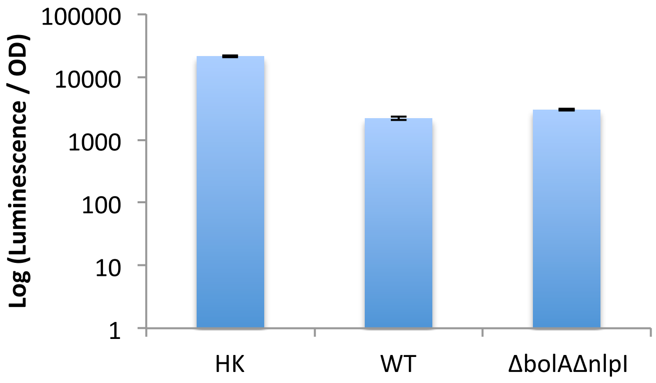

n=2

n=2, t=0: Act

ΔdsbA, 37°C

Growth, n=3

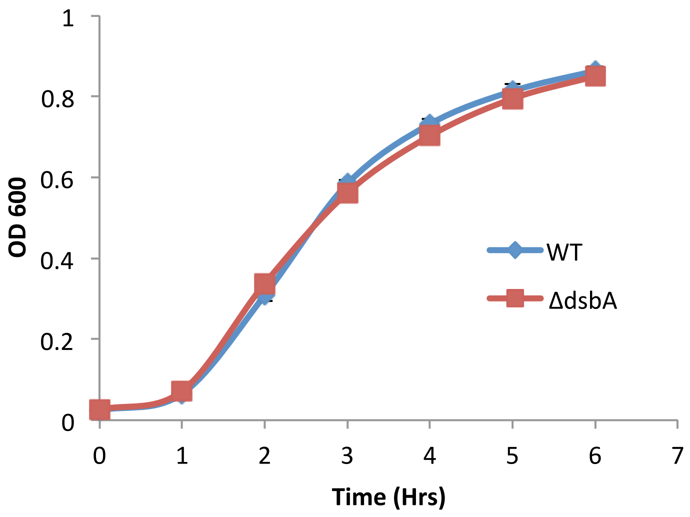

Sytox Green

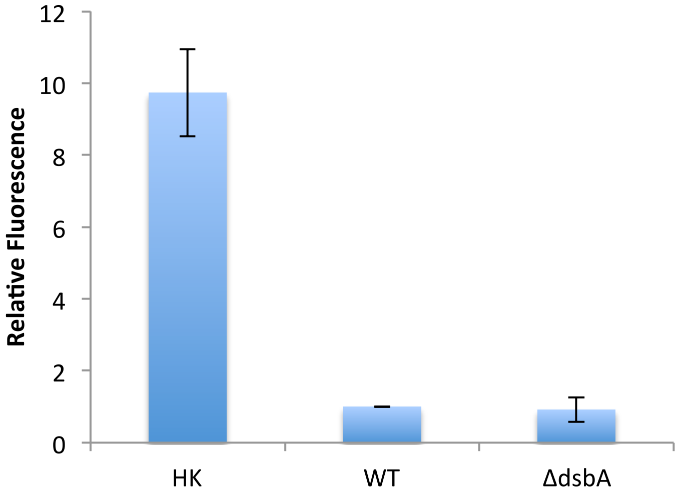

n=2

5 µg/ ml Actinomycin D

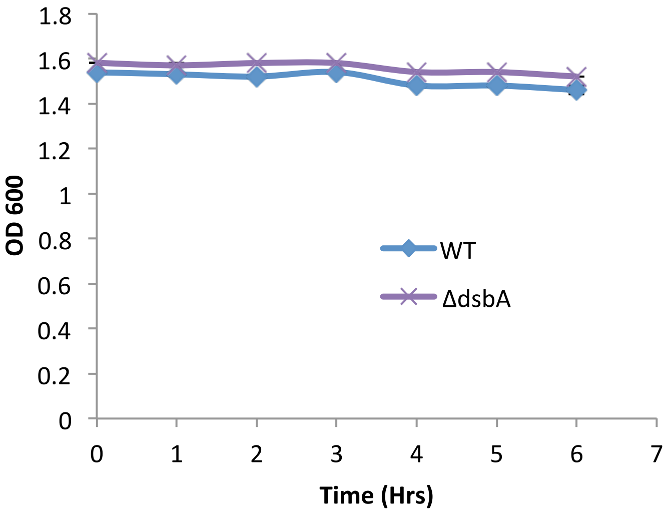

Adenylate Kinase

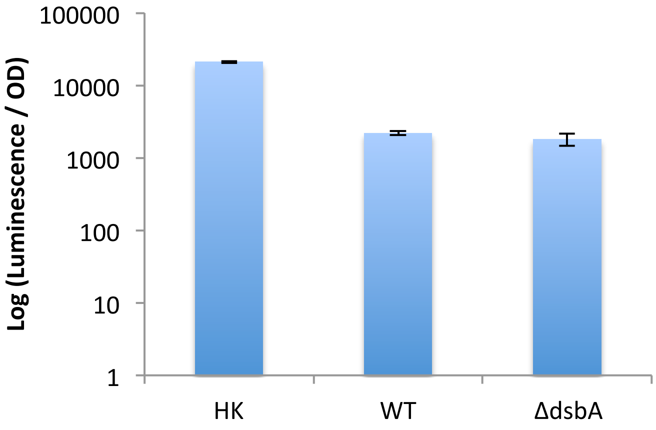

n=2

n=2, t=0: Act

ΔnlpIΔdsbA, 37°C

Growth, n=3

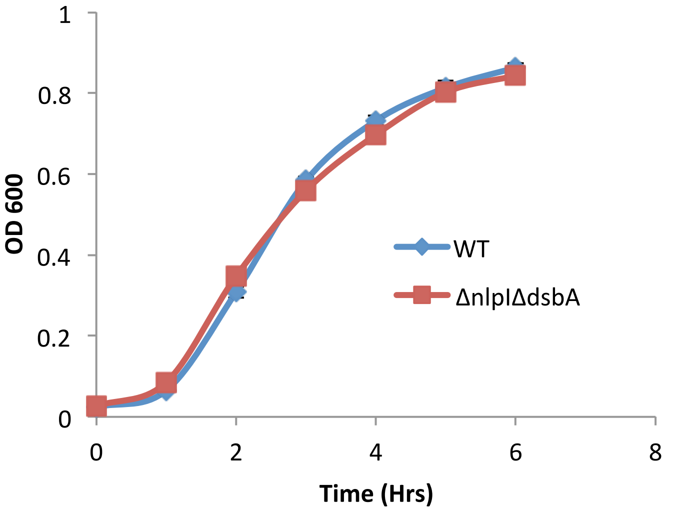

Sytox Green

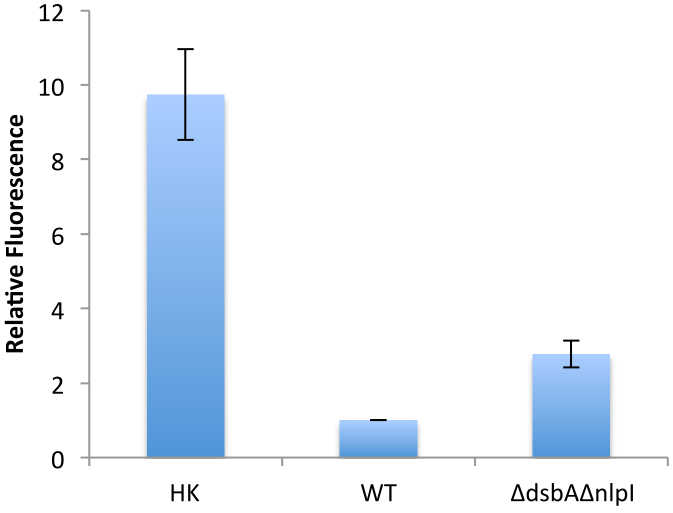

n=2

5 µg/ ml Actinomycin D

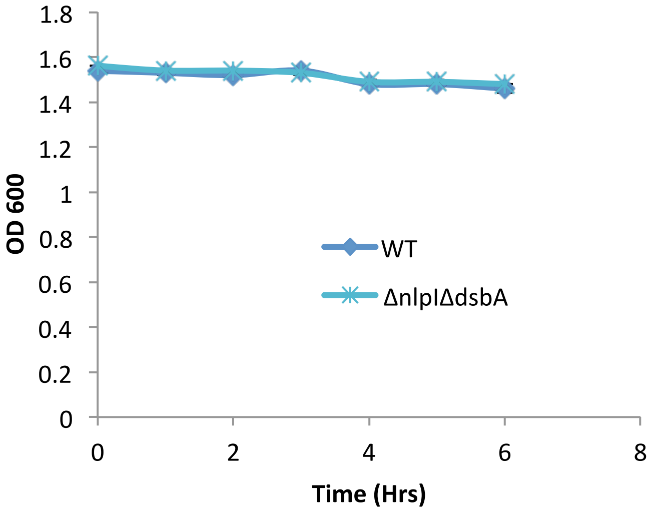

Adenylate Kinase

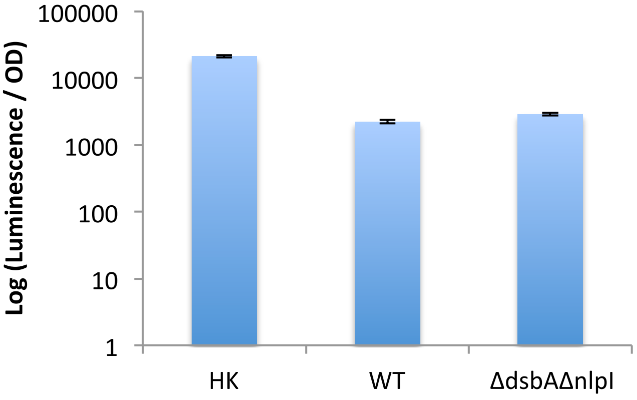

n=2

n=2, t=0: Act

Δspr, 37°C

Growth, n=3

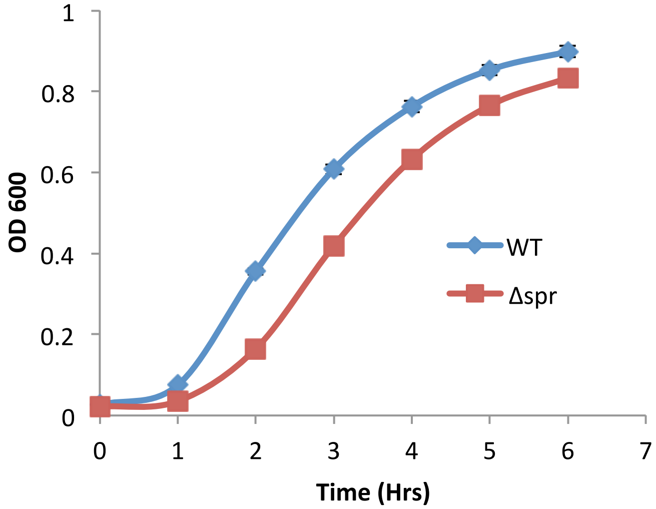

Sytox Green

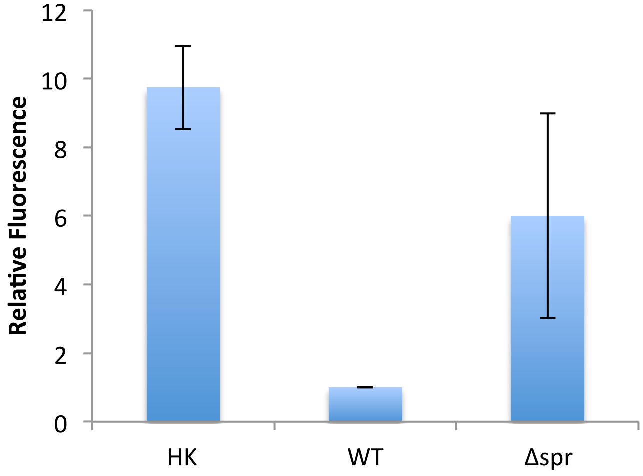

n=2

5 µg/ ml Actinomycin D

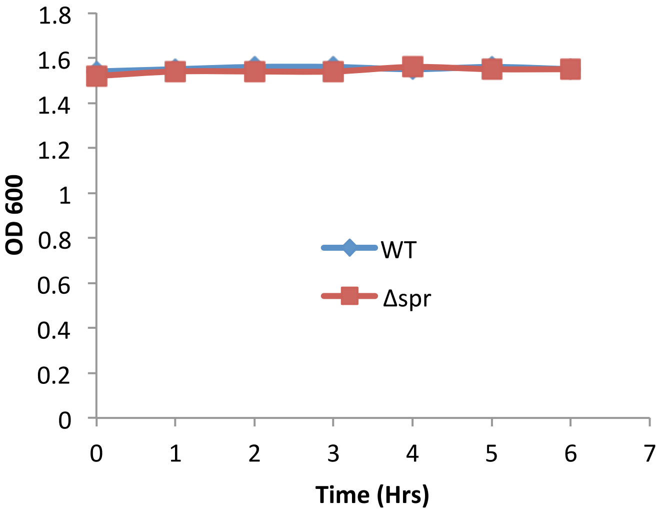

Adenylate Kinase

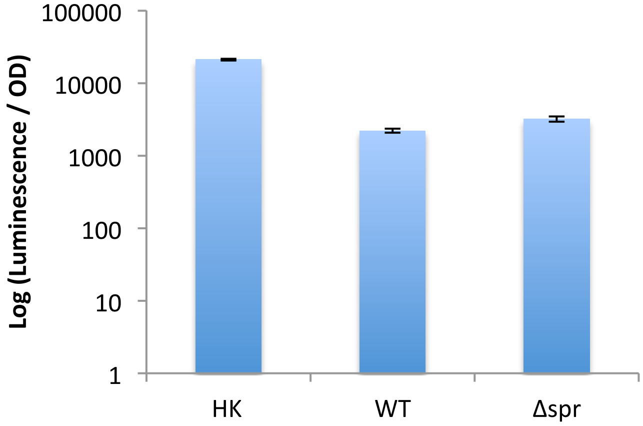

n=2

n=2, t=0: Act

ΔnlpIΔspr, 37°C

Growth, n=3

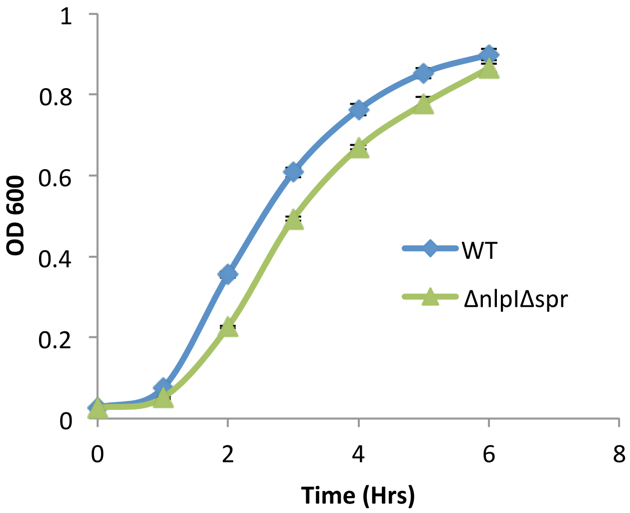

Sytox Green

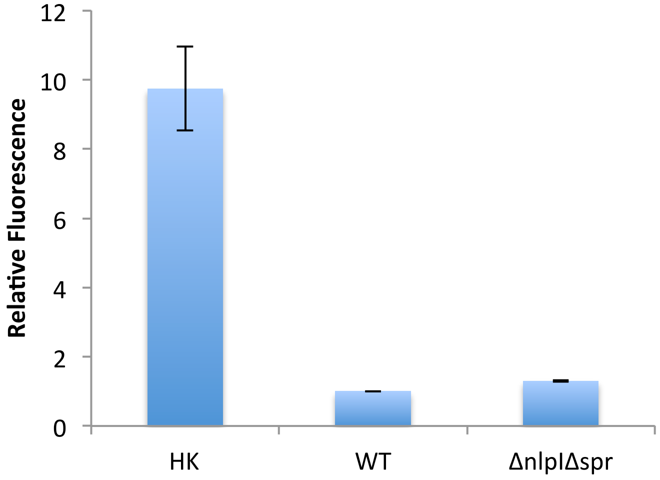

n=2

5 µg/ ml Actinomycin D

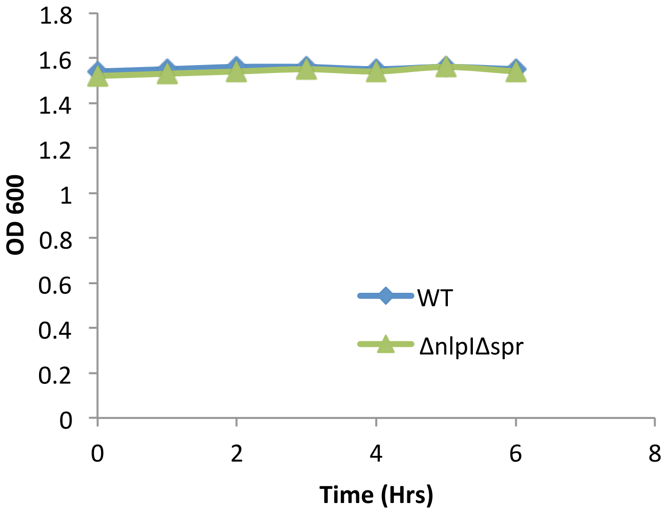

Adenylate Kinase

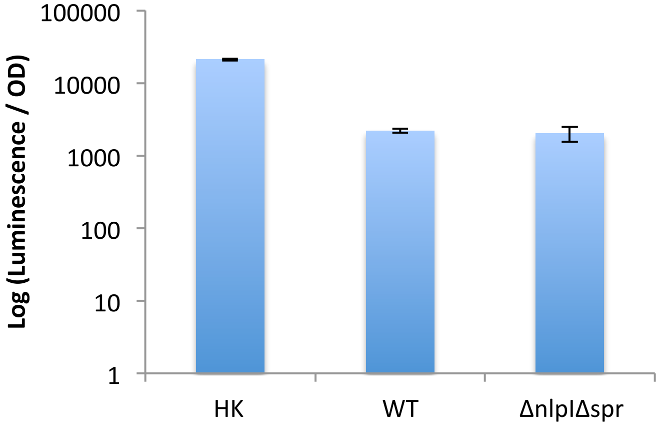

n=2

n=2, t=0: Act

ΔnlpIΔnlpC, 37°C

Growth, n=3

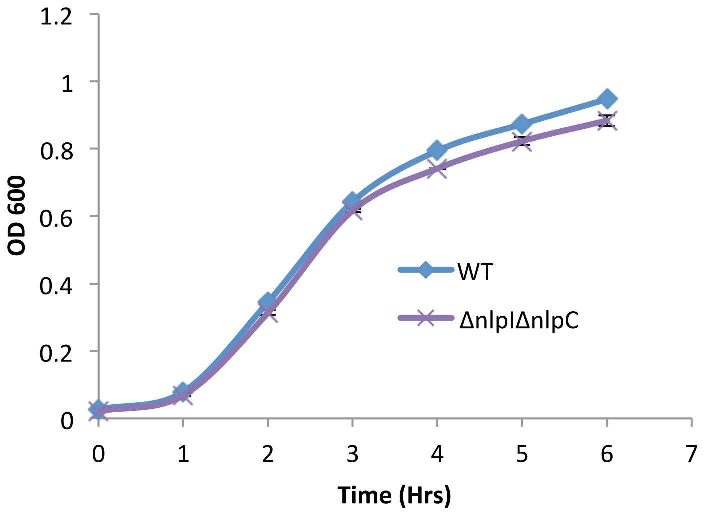

Sytox Green

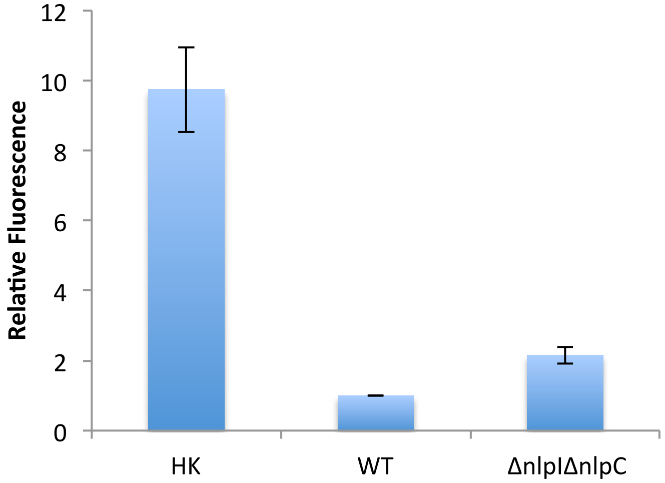

n=2

5 µg/ ml Actinomycin D

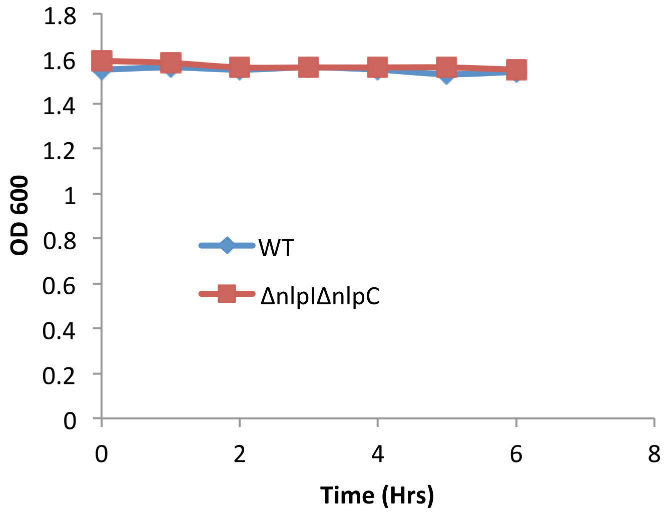

Adenylate Kinase

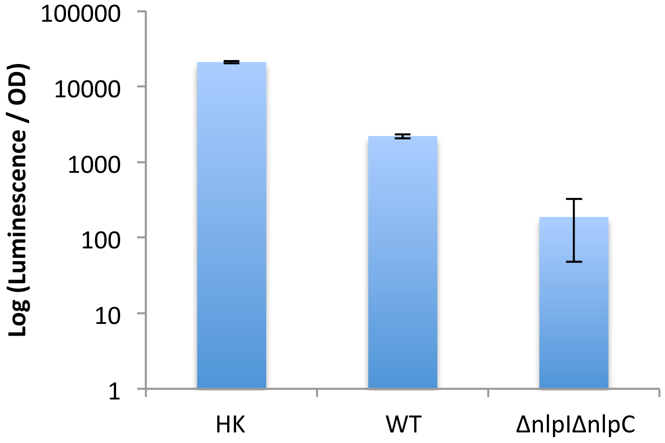

n=2

n=2, t=0: Act

ΔnlpIΔydhO, 37°C

Growth, n=3

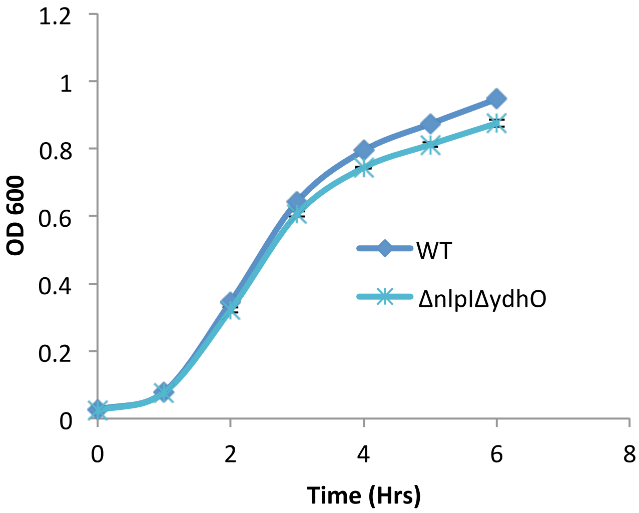

Sytox Green

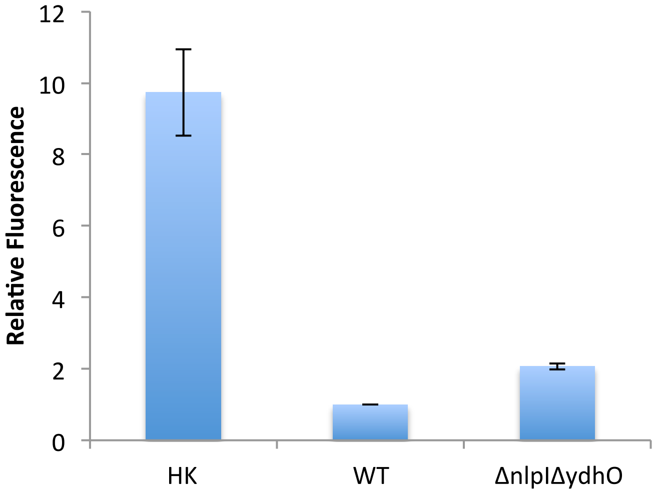

n=2

5 µg/ ml Actinomycin D

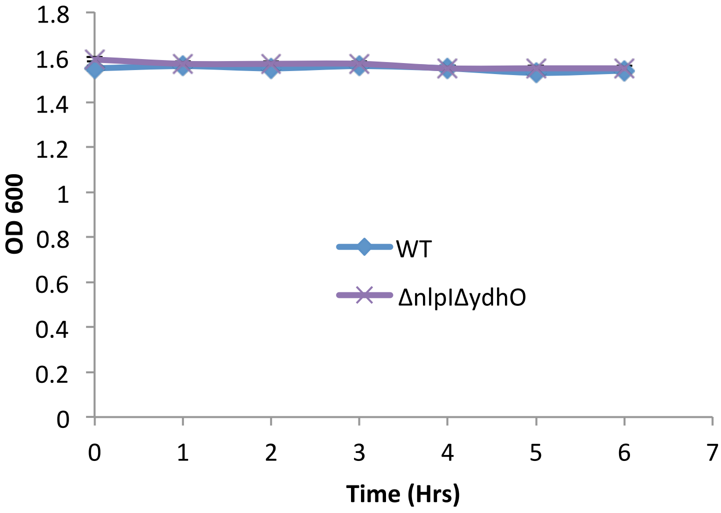

n=2, t=0: Act

Adenylate Kinase

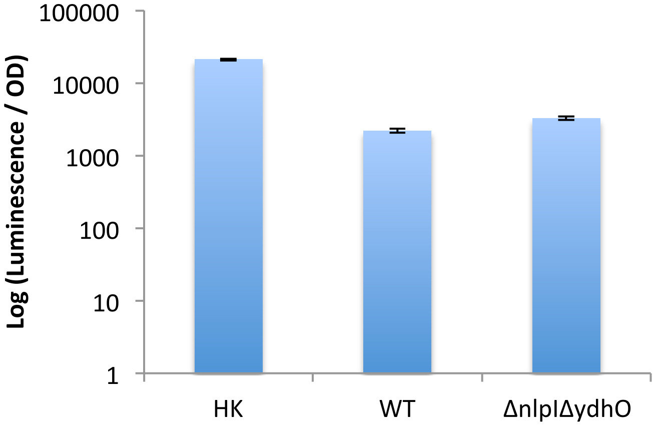

n=2

ΔnlpIΔyafL, 37°C

Growth, n=3

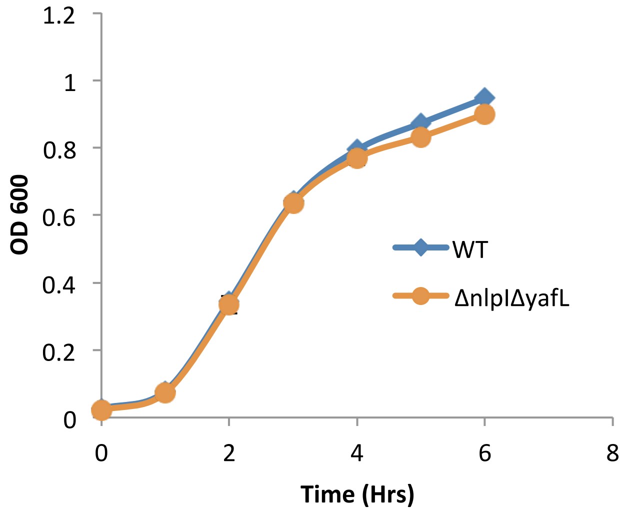

Sytox Green

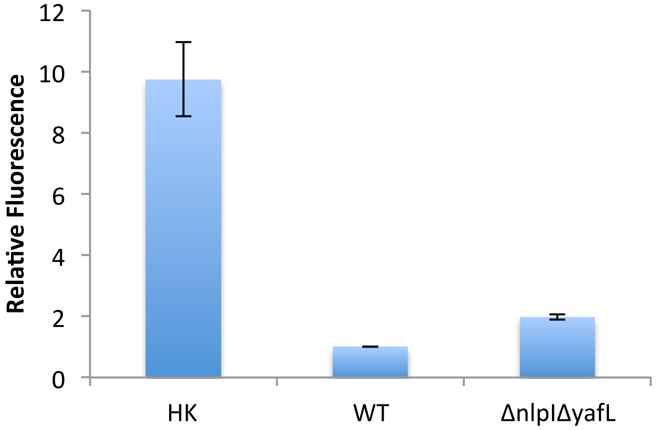

n=2

5 µg/ ml Actinomycin D

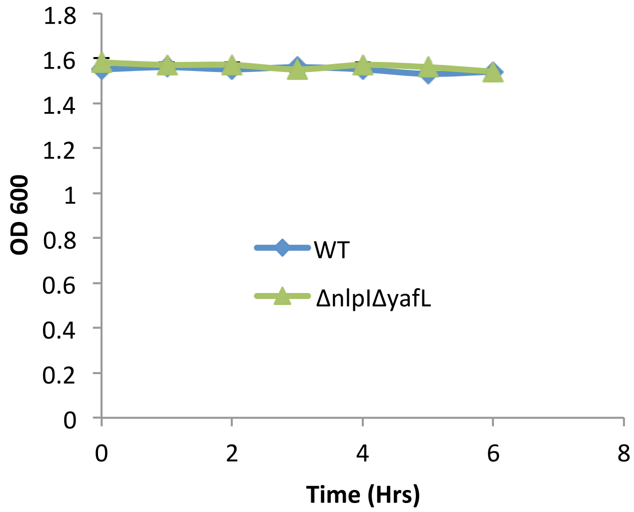

Adenylate Kinase

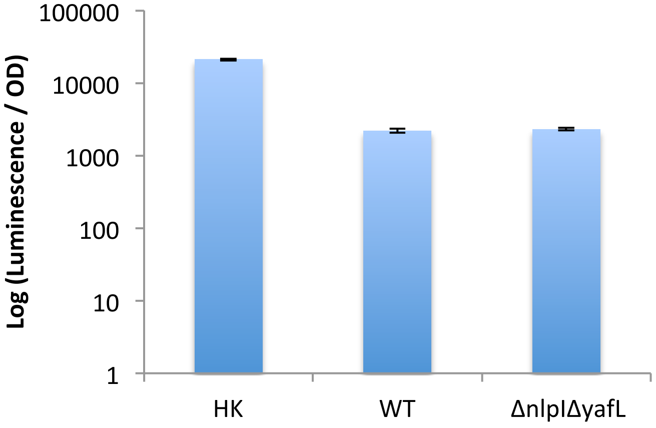

n=2

n=2, t=0: Act

ΔnlpIΔyebA, 37°C

Growth, n=3

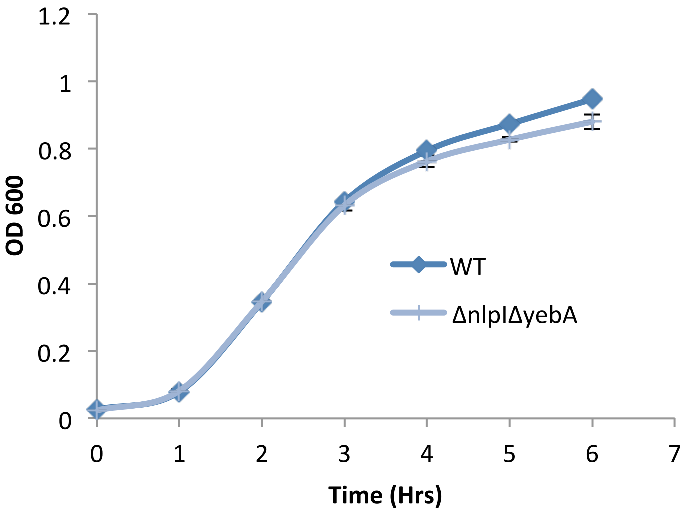

Sytox Green

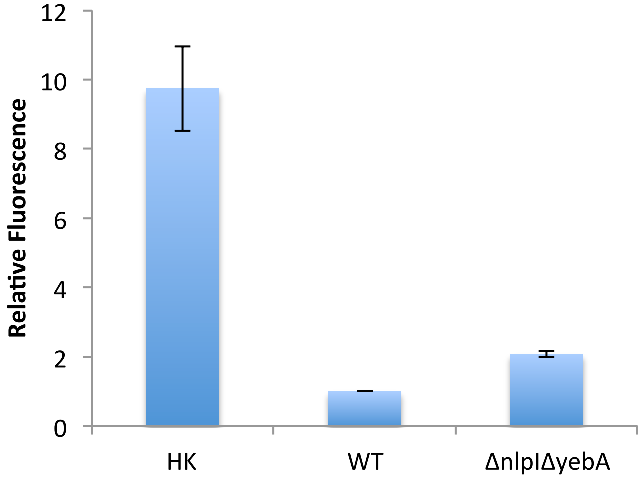

n=2

5 µg/ ml Actinomycin D

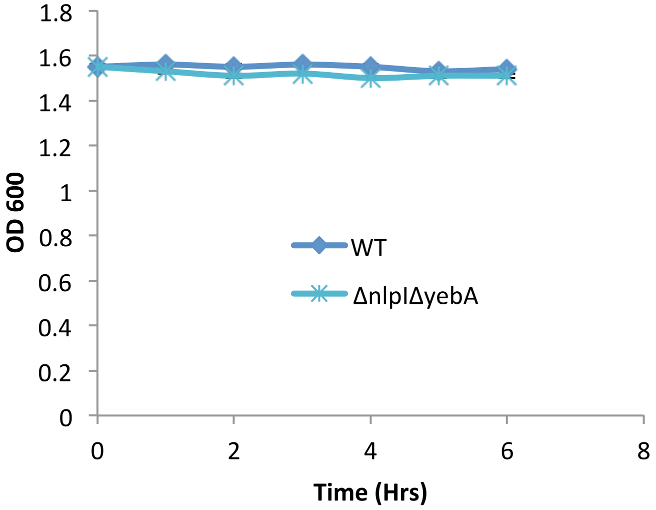

Adenylate Kinase

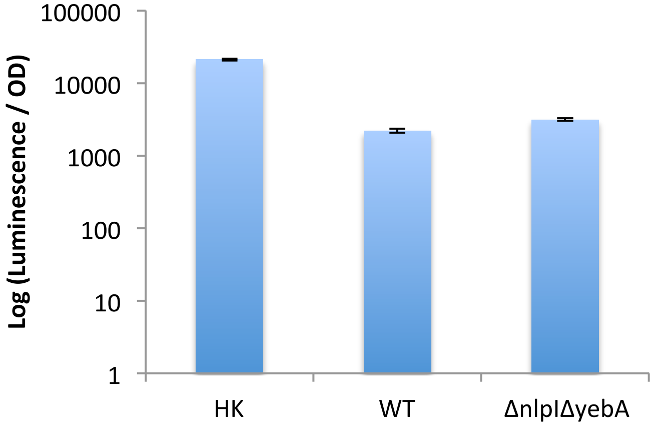

n=2

n=2, t=0: Act

ΔdegP, 37°C

Growth, n=3

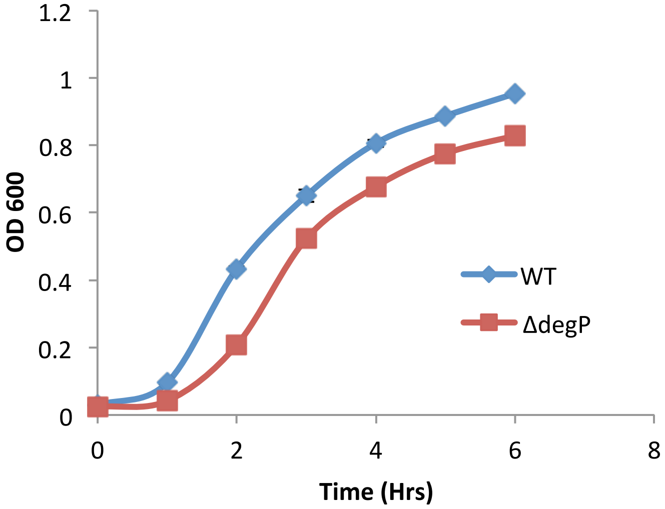

Sytox Green

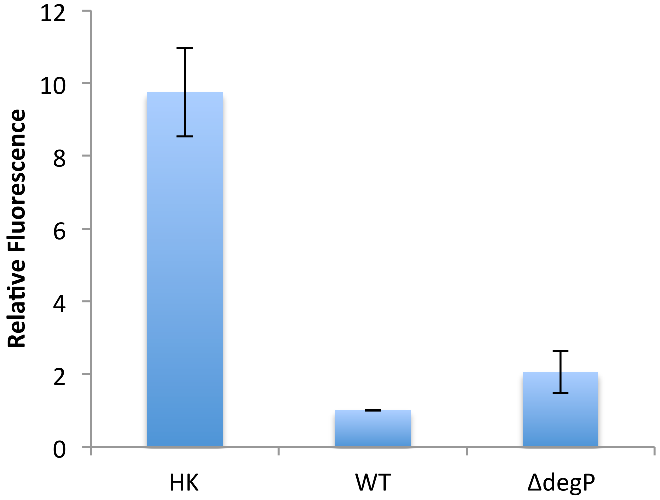

n=2

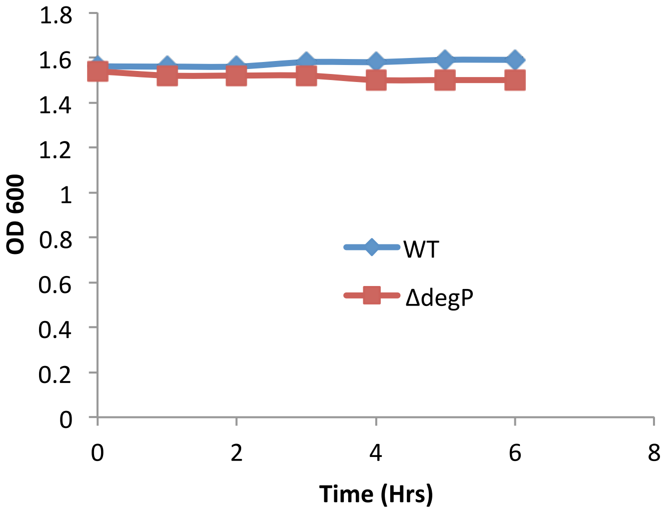

Adenylate Kinase

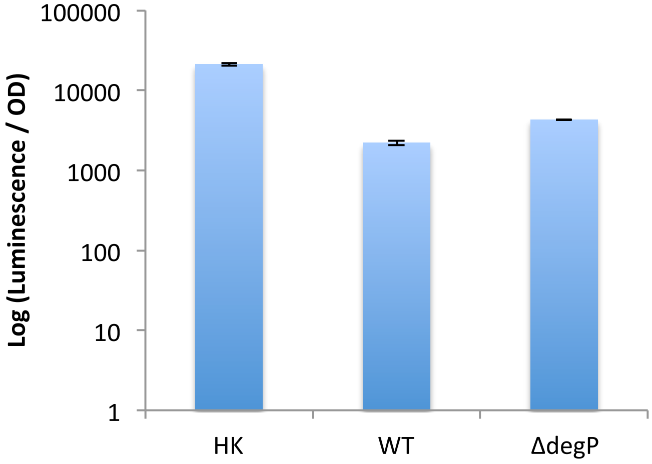

n=2

n=2, t=0: Act

ΔsprΔdegP, 37°C

Growth, n=3

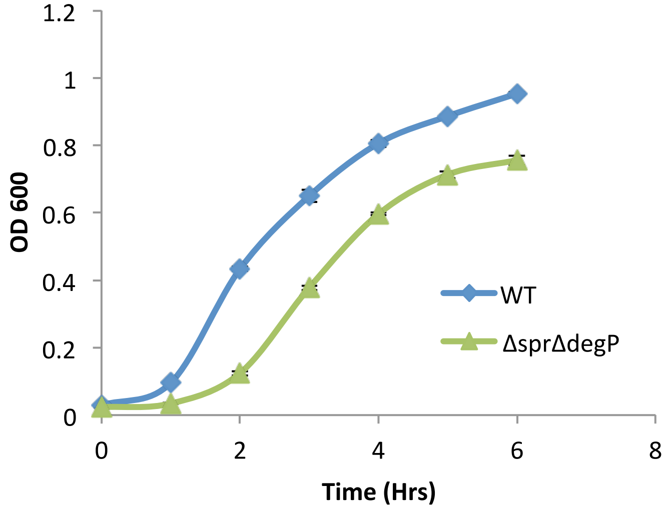

Sytox Green

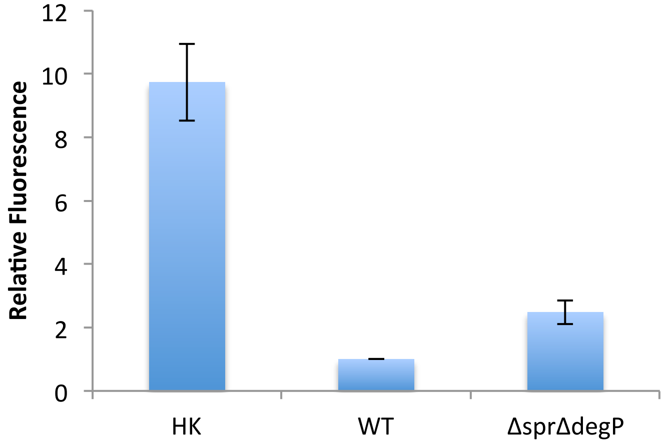

n=2

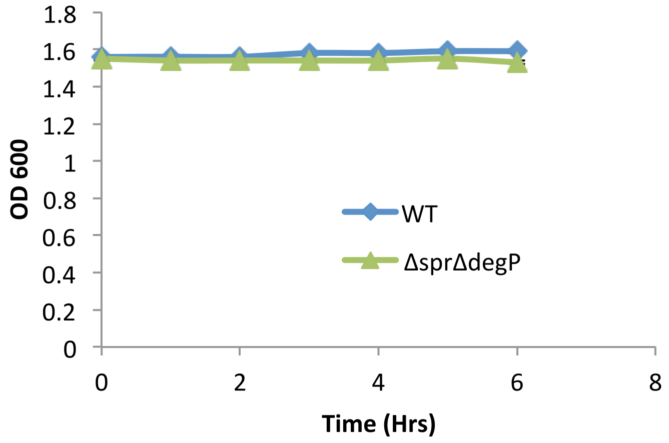

Adenylate Kinase

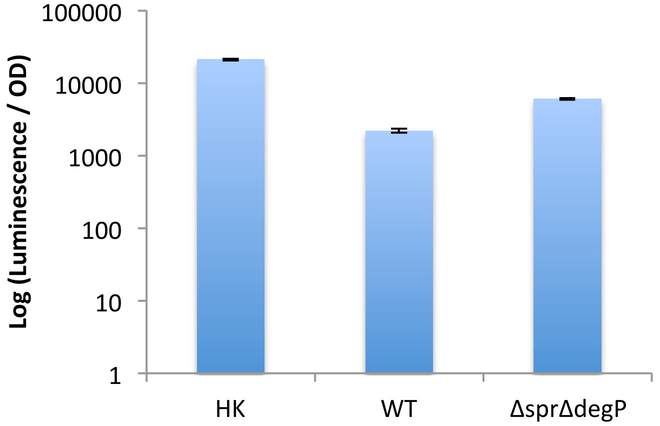

n=2

n=2, t=0: Act

ΔnlpIΔsprΔdegP, 37°C

Growth, n=3

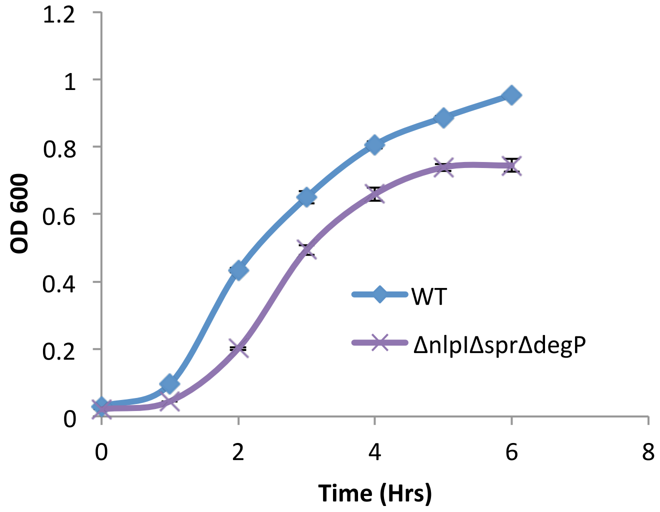

Sytox Green

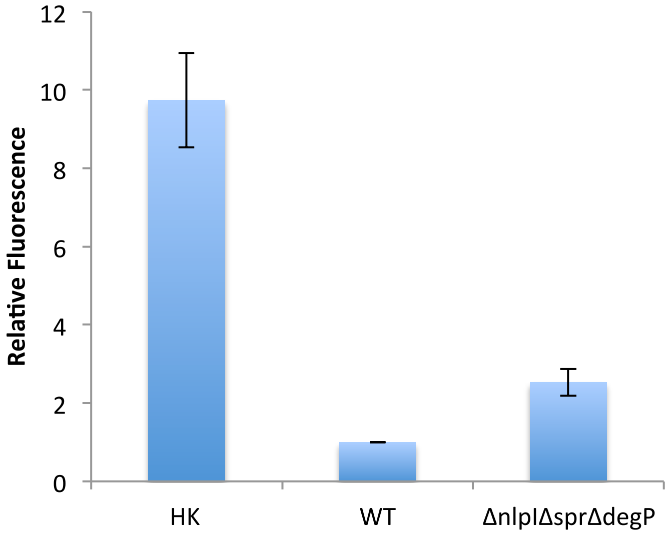

n=2

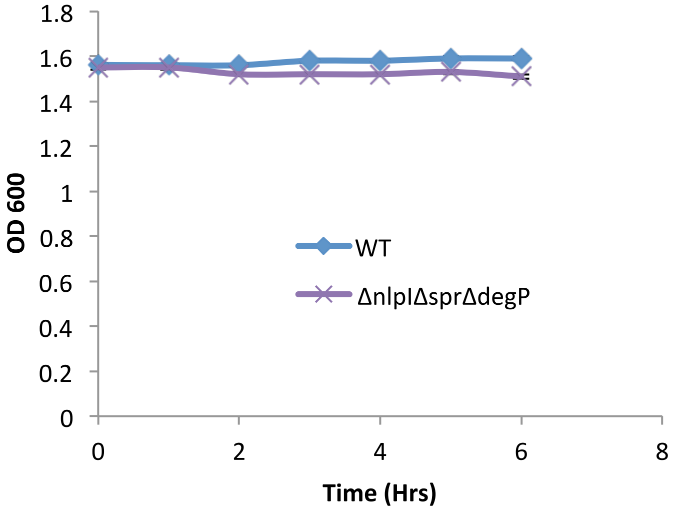

Adenylate Kinase

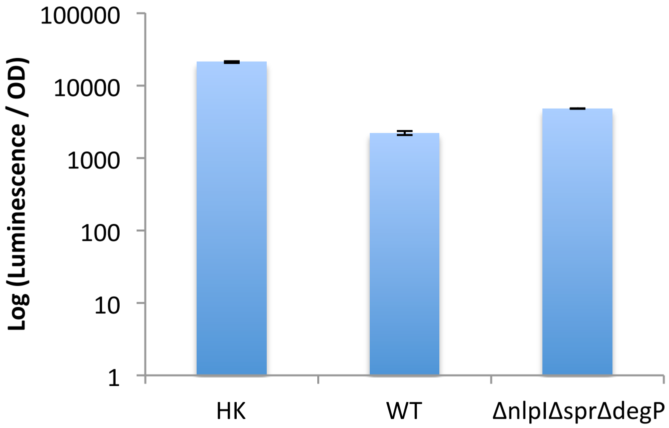

n=2

n=2, t=0: Act
